# Supplementary material for: Applicability of the ReproQ client experiences questionnaire for quality improvement in maternity care
Source: PeerJ. 2016 Jul 13;4:e2092. doi: 10.7717/peerj.2092 (PMC4950561; doi:10.7717/peerj.2092)
Supplement: File S1 [file peerj-04-2092-s001.docx]

Supplementary file 1A. Difference in ReproQ in terms of negative score, between the least preferred and the most preferred state, in 6 known-groups (n=4883)

|  |  | Negative experience - individual selection | | | | | | | | | | |
| --- | --- | --- | --- | --- | --- | --- | --- | --- | --- | --- | --- | --- |
|  | N | TT | PS | ST | DI | AU | CF | CM | PA | SC | BA | CC |
| **Professional continuity** |  |  |  |  |  |  |  |  |  |  |  |  |
| Yes | 2541 | 37.4 | 23.6 | 20.4 | 3.2 | 19.6 | 2.8 | 1.9 | 6.5 | 1.4 | 2.2 | 13.3 |
| No | 2313 | 53.7 | 39.4 | 31.6 | 8.8 | 32.0 | 5.2 | 5.0 | 8.3 | 3.5 | 3.3 | 24.0 |
| *Difference* |  | 16.3^7, 9^ | 15.8^7, 9^ | 11.2^9^ | 5.6^9^ | 12.4^7, 9^ | 2.4^7, 9^ | 3.1^7, 9^ | 1.8^9^ | 2.1^7, 9^ | 1.1^9^ | 10.7^7, 9^ |
| **Setting continuity** |  |  |  |  |  |  |  |  |  |  |  |  |
| Primary care only | 1773 | 31.8 | 18.3 | 18.6 | 1.7 | 15.6 | 2.0 | 0.8 | 7.8 | 0.7 | 1.5 | 10.8 |
| Secondary care only | 738 | 54.7 | 41.6 | 29.1 | 8.5 | 35.1 | 5.7 | 5.7 | 6.4 | 3.5 | 5.0 | 21.8 |
| Referred during pregnancy | 527 | 54.1 | 41.7 | 29.8 | 7.8 | 35.3 | 3.4 | 3.8 | 6.1 | 3.4 | 4.2 | 23.5 |
| Referred during parturition | 1724 | 52.4 | 41.7 | 30.9 | 8.4 | 28.9 | 5.5 | 5.2 | 7.9 | 3.3 | 2.6 | 23.7 |
| *Difference* |  | 20.6^7, 9^ | 23.4^7, 9^ | 12.3^7, 9^ | 6.7^9^ | 13.3^7, 9^ | 3.5^7, 9^ | 4.4^7, 9^ | 0.1^9^ | 2.6^7, 9^ | 1.1^9^ | 12.9^7, 9^ |
| **Onset of delivery** |  |  |  |  |  |  |  |  |  |  |  |  |
| In office hours | 1492 | 49.3 | 33.8 | 28.7 | 6.9 | 27.7 | 4.8 | 4.0 | 7.0 | 3.6 | 4.3 | 20.3 |
| Outside office hours | 3357 | 43.4 | 30.0 | 24.5 | 5.4 | 24.6 | 3.6 | 3.2 | 7.6 | 1.8 | 2.0 | 17.6 |
| *Difference* |  | 5.9 | 3.8 | 4.2 | 1.5 | 3.1 | 1.2 | 0.8 | -0.6 | 1.8^7, 9^ | 2.3^9^ | 2.7 |
| **Cesarean section** |  |  |  |  |  |  |  |  |  |  |  |  |
| Planned | 214 | 57.0 | 42.5 | 28.0 | 7.9 | 36.0 | 7.9 | 7.0 | 3.3 | 3.3 | 10.7 | 21.0 |
| Emergency | 404 | 55.9 | 43.1 | 29.5 | 10.1 | 33.4 | 6.2 | 8.7 | 5.4 | 3.2 | 5.2 | 23.0 |
| *Difference* |  | -1.1 | 0.6 | 1.5 | 2.2 | -2.6 | -1.7 | 1.7 | 2.1^9^ | -0.1 | -5.5^7, 9^ | 2.0 |
| **Travel time** |  |  |  |  |  |  |  |  |  |  |  |  |
| < 15 minutes | 827 | 40.0 | 27.4 | 22.9 | 5.1 | 20.5 | 4.7 | 3.0 | 6.2 | 2.5 | 2.5 | 16.4 |
| ≥15 minutes | 549 | 45.5 | 32.0 | 25.8 | 7.5 | 26.5 | 4.9 | 3.6 | 8.2 | 2.7 | 2.5 | 18.7 |
| *Difference* |  | 5.5 | 4.6 | 2.9 | 2.4 | 6.0^7, 9^ | 0.2 | 0.6 | 2.0^7, 9^ | 0.2 | 0.0 | 2.3 |
| **Hospital size** |  |  |  |  |  |  |  |  |  |  |  |  |
| <750 | 159 | 51.6 | 35.2 | 30.8 | 10.7 | 28.3 | 3.1 | 7.5 | 7.5 | 3.8 | 5.7 | 20.8 |
| ≥1500 | 604 | 52.2 | 38.1 | 27.0 | 6.1 | 31.5 | 4.5 | 3.5 | 4.8 | 2.0 | 3.5 | 21.0 |
| *Difference* |  | -0.6 | -2.9 | 3.8 | 4.6^9^ | -3.2 | -1.4 | 4.0^7, 9^ | 2.7^9^ | 1.8^7, 9^ | 2.2^9^ | -0.2 |

Supplementary file 1B. Difference in ReproQ in terms of mean score, between the least preferred and the most preferred state, in 6 known-groups (n=4883)

|  |  | Mean experience | | | | | | | | | | |
| --- | --- | --- | --- | --- | --- | --- | --- | --- | --- | --- | --- | --- |
|  | N | TT | PS | ST | DI | AU | CF | CM | PA | SC | BA | CC |
| **Professional continuity** |  |  |  |  |  |  |  |  |  |  |  |  |
| Yes | 2541 | 3.81 | 3.78 | 3.83 | 3.89 | 3.58 | 3.84 | 3.79 | 3.83 | 3.89 | 3.89 | 3.77 |
| No | 2313 | 3.67 | 3.66 | 3.72 | 3.75 | 3.34 | 3.74 | 3.66 | 3.69 | 3.81 | 3.84 | 3.53 |
| *Difference* |  | 0.14^7, 9^ | 0.12^9^ | 0.10^9^ | 0.13^9^ | 0.25^7, 9^ | 0.10^9^ | 0.13^9^ | 0.14^7, 9^ | 0.08^9^ | 0.05^9^ | 0.23^7^ |
| **Setting continuity** |  |  |  |  |  |  |  |  |  |  |  |  |
| Primary care only | 1773 | 3.85 | 3.82 | 3.88 | 3.93 | 3.68 | 3.87 | 3.83 | 3.86 | 3.92 | 3.91 | 3.82 |
| Secondary care only | 738 | 3.67 | 3.60 | 3.72 | 3.76 | 3.28 | 3.71 | 3.66 | 3.71 | 3.80 | 3.82 | 3.56 |
| Referred during pregnancy | 527 | 3.71 | 3.66 | 3.74 | 3.81 | 3.30 | 3.80 | 3.72 | 3.74 | 3.82 | 3.83 | 3.57 |
| Referred during parturition | 1724 | 3.68 | 3.63 | 3.73 | 3.75 | 3.36 | 3.73 | 3.64 | 3.70 | 3.82 | 3.86 | 3.55 |
| *Difference* |  | 0.18^7^ | 0.20^7, 9^ | 0.14^7, 9^ | 0.18^7, 9^ | 0.32^7, 9^ | 0.14^9^ | 0.19^7, 9^ | 0.16^7, 9^ | 0.10^9^ | 0.05^9^ | 0.26^7, 9^ |
| **Onset of delivery** |  |  |  |  |  |  |  |  |  |  |  |  |
| In office hours | 1492 | 3.73 | 3.69 | 3.75 | 3.81 | 3.42 | 3.77 | 3.71 | 3.75 | 3.84 | 3.85 | 3.63 |
| Outside office hours | 3357 | 3.75 | 3.73 | 3.79 | 3.83 | 3.48 | 3.79 | 3.74 | 3.77 | 3.86 | 3.88 | 3.67 |
| *Difference* |  | 0.03 | 0.04 | 0.03 | 0.02 | 0.06 | 0.02 | 0.03 | 0.02 | 0.03 | 0.02 | 0.04 |
| **Cesarean section** |  |  |  |  |  |  |  |  |  |  |  |  |
| Planned | 214 | 3.72 | 3.61 | 3.71 | 3.84 | 3.12 | 3.71 | 3.71 | 3.83 | 3.84 | 3.80 | 3.67 |
| Emergency | 404 | 3.64 | 3.56 | 3.67 | 3.70 | 3.22 | 3.69 | 3.62 | 3.72 | 3.79 | 3.79 | 3.51 |
| *Difference* |  | 0.07 | 0.05 | 0.04 | 0.14^9^ | -0.10 | 0.03 | 0.09 | -0.11^9^ | 0.04^9^ | 0.01 | 0.16^9^ |
| **Travel time** |  |  |  |  |  |  |  |  |  |  |  |  |
| < 15 minutes | 827 | 3.74 | 3.69 | 3.79 | 3.81 | 3.50 | 3.76 | 3.70 | 3.78 | 3.85 | 3.87 | 3.67 |
| ≥15 minutes | 549 | 3.72 | 3.68 | 3.77 | 3.78 | 3.43 | 3.76 | 3.70 | 3.73 | 3.84 | 3.87 | 3.63 |
| *Difference* |  | 0.02 | 0.02 | 0.03 | 0.03 | 0.06 | 0.00 | 0.00 | 0.05 | 0.01 | 0.00 | 0.05 |
| **Hospital size** |  |  |  |  |  |  |  |  |  |  |  |  |
| <750 | 159 | 3.69 | 3.65 | 3.70 | 3.78 | 3.34 | 3.81 | 3.66 | 3.72 | 3.81 | 3.76 | 3.57 |
| ≥1500 | 604 | 3.72 | 3.66 | 3.74 | 3.81 | 3.35 | 3.78 | 3.72 | 3.76 | 3.85 | 3.88 | 3.59 |
| *Difference* |  | 0.03 | 0.01 | 0.04 | 0.03 | 0.00 | -0.04 | 0.06 | 0.04 | 0.04^9^ | 0.117^7, 9^ | 0.01 |
