# Supplementary figures and images for: Applicability of the ReproQ client experiences questionnaire for quality improvement in maternity care
Source: PeerJ. 2016 Jul 13;4:e2092. doi: 10.7717/peerj.2092 (PMC4950561; doi:10.7717/peerj.2092)

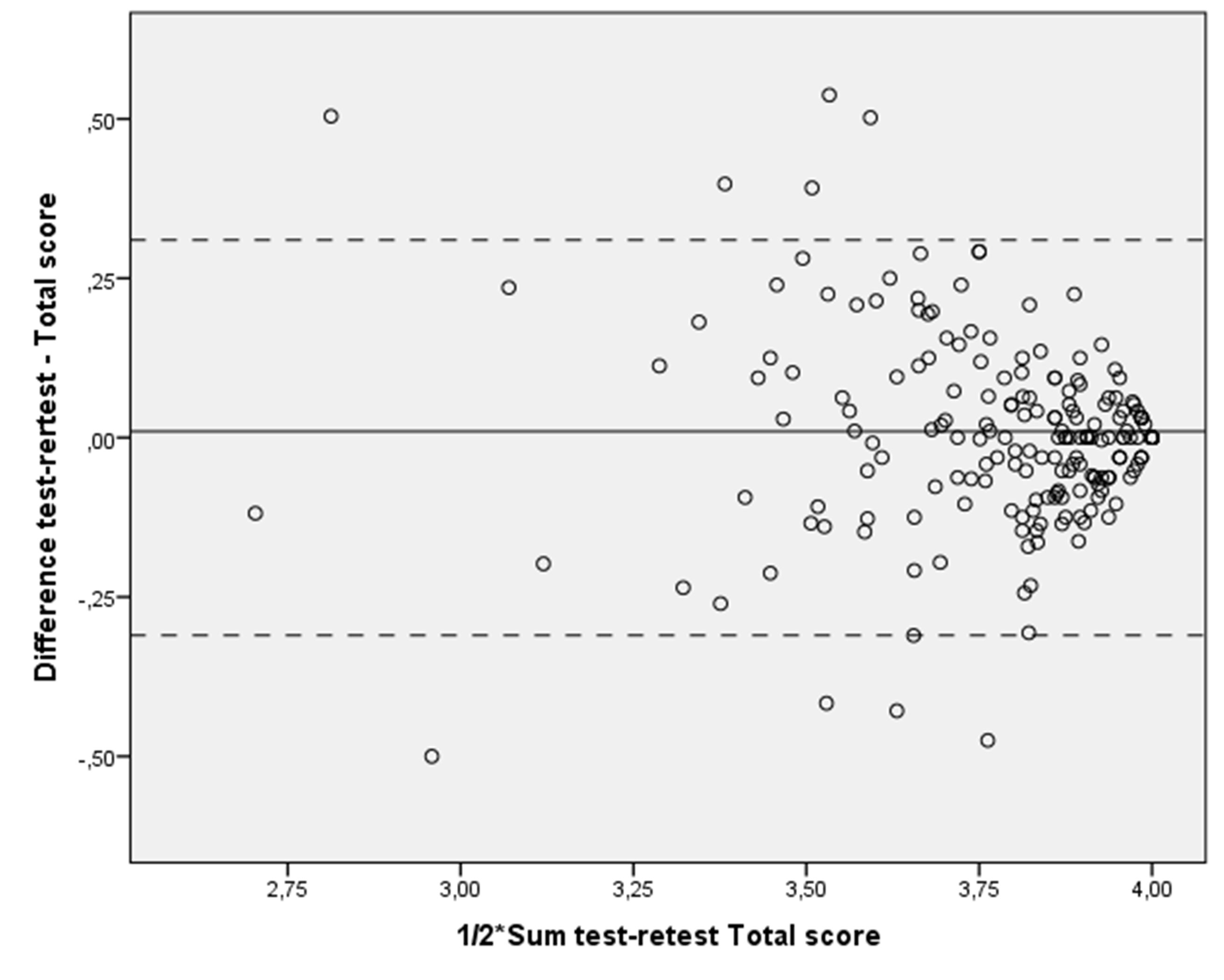

Supplement: File S2 [file peerj-04-2092-s002.tif]
