# Supplementary material for: Applicability of the ReproQ client experiences questionnaire for quality improvement in maternity care
Source: PeerJ. 2016 Jul 13;4:e2092. doi: 10.7717/peerj.2092 (PMC4950561; doi:10.7717/peerj.2092)
Supplement: Supplemental Information 1 [file peerj-04-2092-s003.docx]

Code

# CHARACTERITISCS.

*IEDEREEN.

FREQUENCIES SD_Lft_S_Cat4 SD_Etm_S_Cat2 SD_Opl_S_Cat3 SD_Rel_S EE_Par_S_Cat3 OV_All_S_Cat5 Zorgproces_S_Cat4 ZB_Kjn_S_Cat3 OV_Kza_S_Cat2 ZB_Urb_S ZB_Kan_S_Cat2 ZB_Ret_S_Cat2 VSV_GR_S.

FREQUENCIES SD_Lft_M_Cat4 SD_Etm_M_Cat2 SD_Opl_M_Cat3 SD_Rel_M EE_Par_M_Cat3 OV_All_M_Cat5 Zorgproces_M_Cat4 ZB_Kjn_M_Cat3 OV_Kza_M_Cat2 ZB_Urb_M ZB_Kan_M_Cat2 ZB_Ret_M_Cat2 VSV_GR_M.

FREQUENCIES SD_Lft_S

/STATISTICS MEAN STDDEV.

*ALLEEN TEST.

* aanmaken variabele die test van hertest respondenten scheid.

NUMERIC TH_Res (F2.0).

COMPUTE TH_Res = -999.

IF (TH_Ver_S_D=0) TH_Res = 2.

IF SYSMIS(TH_Ver_S_D) TH_Res = 1.

VARIABLE LABELS TH_Res 'bepaling welke respondenten test en/of hertest hebben ingevuld'.

VALUE LABELS TH_Res

1 'test'

2 'test en hertest'.

FREQUENCIES TH_Res.

* STAP 2: karakteristieken van alleen hertest respondenten.

* Aanmaken filter.

NUMERIC F_T_Res (F2.0).

COMPUTE F_T_Res = $SYSMIS.

IF (TH_Res = 1) F_T_Res = 1.

VARIABLE LABELS F_T_Res 'vrouwen die alleen de test hebben ingevuld'.

VALUE LABELS F_T_Res

1 'test'.

FREQUENCIES F_T_Res.

SORT CASES BY F_T_Res.

FILTER BY F_T_Res.

FREQUENCIES SD_Lft_S_Cat4 SD_Etm_S_Cat2 SD_Opl_S_Cat3 SD_Rel_S EE_Par_S_Cat3 OV_All_S_Cat5 Zorgproces_S_Cat4

ZB_Kjn_S_Cat3 OV_Kza_S_Cat2 ZB_Urb_S ZB_Kan_S_Cat2 ZB_Ret_S_Cat2 VSV_GR_S.

FREQUENCIES SD_Lft_M_Cat4 SD_Etm_M_Cat2 SD_Opl_M_Cat3 SD_Rel_M EE_Par_M_Cat3 OV_All_M_Cat5 Zorgproces_M_Cat4

ZB_Kjn_M_Cat3 OV_Kza_M_Cat2 ZB_Urb_M ZB_Kan_M_Cat2 ZB_Ret_M_Cat2 VSV_GR_M.

FREQUENCIES SD_Lft_S

/STATISTICS MEAN STDDEV.

FILTER OFF.

* STAP 2: karakteristieken van alleen hertest respondenten.

* Aanmaken filter.

NUMERIC F_TH_Res (F2.0).

COMPUTE F_TH_Res = $SYSMIS.

IF (TH_Ver_S_D=0) F_TH_Res = 1.

VARIABLE LABELS F_TH_Res 'vrouwen die hertest hebben ingevuld'.

VALUE LABELS F_TH_Res

1 'test en hertest'.

FREQUENCIES F_TH_Res.

SORT CASES BY F_TH_Res.

FILTER BY F_TH_Res.

FREQUENCIES SD_Lft_S_Cat4 SD_Etm_S_Cat2 SD_Opl_S_Cat3 SD_Rel_S EE_Par_S_Cat3 OV_All_S_Cat5 Zorgproces_S_Cat4

ZB_Kjn_S_Cat3 OV_Kza_S_Cat2 ZB_Urb_S ZB_Kan_S_Cat2 ZB_Ret_M_Cat2 VSV_GR_S.

FREQUENCIES SD_Lft_M_Cat4 SD_Etm_M_Cat2 SD_Opl_M_Cat3 SD_Rel_M EE_Par_M_Cat3 OV_All_M_Cat5 Zorgproces_M_Cat4

ZB_Kjn_M_Cat3 OV_Kza_M_Cat2 ZB_Urb_M ZB_Kan_M_Cat2 ZB_Ret_M_Cat2 VSV_GR_M.

FILTER OFF.

* STAP 1: zo nodig, nieuwe variabele aanmaken

* leeftijd 1: Submitdate omzetten in dd-mm-yyyy.

*handmatig in variable views.

* leeftijd 2: Aanpassen variabele SENT.

* Aanmaken stringvariabelen voor elk deel van de datum afzonderlijk: dag, maand, jaar.

* Dag 2 digits.

* Maand 2 digits.

* Jaar 4 digits.

STRING Da_Dag (A2).

STRING Da_Mnd (A2).

STRING Da_Yea (A4).

EXECUTE.

* leeftijd 2B: Digits knippen uit variabele SENT. Geknipte stukjes toewijzen aan nieuwe variablen.

*1. variabele naar waar je uit wil knippen. 2. vanaf welk karakter je wil knippen. 3. aantal karakters dat je wil knippen.

COMPUTE Da_Yea = substr(sent, 1, 4).

VARIABLE LABELS Da_Yea 'jaar van verzenden'.

COMPUTE Da_Mnd = substr(sent, 6, 2).

VARIABLE LABELS Da_Mnd 'maand van verzenden'.

COMPUTE Da_Dag = substr(sent, 9, 2).

VARIABLE LABELS Da_Dag 'dag van verzenden'.

EXECUTE.

*leeftijd 2C: String variabelen omzetten in numerieke variabelen.

ALTER TYPE Da_Yea (A4=F4.0).

ALTER TYPE Da_Mnd (A2=F2.0).

ALTER TYPE Da_Dag (A2=F2.0).

EXECUTE.

*leeftijd 2D: aanmaken 1 variabele als datum, met vorm notering.

COMPUTE sentdate = date.dmy(Da_Dag, Da_Mnd, Da_Yea).

FORMATS sentdate (adate11).

VARIABLE LABELS sentdate 'datum waarom vragenlijst is verstuurd'.

EXECUTE.

STRING Da_VeS (A8).

COMPUTE Da_VeS=CONCAT(STRING(XDATE.YEAR(submitdate),F4.0),STRING(XDATE.MONTH(submitdate),N2),STRING(XDATE.MDAY(submitdate),N2)).

VARIABLE LABELS Da_VeS 'submite date als string variabele'.

FREQUENCIES Da_VeS.

* leeftijd 2: maand 'knippen' uit submitiedatum string.

STRING Da_IMd (A2).

COMPUTE Da_IMd = substr(Da_VeS, 5, 2).

VARIABLE LABELS Da_IMd 'maand van invullen vragenlijst'.

FREQUENCIES Da_IMd.

* leeftijd 3: Maand van invullen van string naar numeriek.

ALTER TYPE Da_IMd (A2=F2.0).

FREQUENCIES Da_IMd.

* leeftijd 4: bepalen of maand waarin vragenlijst is ingevuld, <= aan juni of >= aan juli --> dichotomiseren.

NUMERIC Da_IMd_Cat2 (F2.0).

COMPUTE Da_IMd_Cat2 = $SYSMIS.

IF (Da_IMd = 1 OR Da_IMd = 2 OR Da_IMd = 3 OR Da_IMd = 4 OR Da_IMd = 5 OR Da_IMd = 6) Da_IMd_Cat2 = 0.

IF (Da_IMd = 7 OR Da_IMd = 8 OR Da_IMd = 9 OR Da_IMd = 10 OR Da_IMd = 11 OR Da_IMd = 12) Da_IMd_Cat2 = 1.

VARIABLE LABELS Da_IMd_Cat2 'maand van invullen vragenlijst - cat2'.

FREQUENCIES Da_IMd_Cat2.

* leeftijd 5: Bepalen leeftijd.

NUMERIC SD_Lft_S (F4.0).

COMPUTE SD_Lft_S= -999.

IF (Da_IMd_Cat2 = 0) SD_Lft_S = 2014 - SD_Gjr_M - 1.

IF (Da_IMd_Cat2 = 1) SD_Lft_S = 2014 - SD_Gjr_M.

IF (SYSMIS(Da_IMd_Cat2)) SD_Lft_S = 2014 - SD_Gjr_M.

VARIABLE LABELS SD_Lft_S 'leeftijd'.

FREQUENCIES SD_Lft_S .

* leeftijd 6: leeftijdcategorieen: <=25; 26-30; 31-35; >=36.

NUMERIC SD_Lft_S_Cat4 (F2.0).

COMPUTE SD_Lft_S_Cat4 =999.

IF (SD_Lft_S=14 OR SD_Lft_S=15 OR SD_Lft_S=16 OR SD_Lft_S=17 OR SD_Lft_S=18 OR SD_Lft_S=19 OR

SD_Lft_S=20 OR SD_Lft_S=21 OR SD_Lft_S=22 OR SD_Lft_S=23 OR SD_Lft_S=24) SD_Lft_S_Cat4= 1.

IF (SD_Lft_S=25 OR SD_Lft_S=26 OR SD_Lft_S=27 OR SD_Lft_S=28 OR SD_Lft_S=29) SD_Lft_S_Cat4= 2.

IF (SD_Lft_S=30 OR SD_Lft_S=31 OR SD_Lft_S=32 OR SD_Lft_S=33 OR SD_Lft_S=34 ) SD_Lft_S_Cat4= 3.

IF (SD_Lft_S=35 OR SD_Lft_S=36 OR SD_Lft_S=37 OR SD_Lft_S=38 OR SD_Lft_S=39 OR SD_Lft_S=40 OR

SD_Lft_S=41 OR SD_Lft_S=42 OR SD_Lft_S=43 OR SD_Lft_S=44 OR SD_Lft_S=45 ) SD_Lft_S_Cat4= 4.

VARIABLE LABELS SD_Lft_S_Cat4 'leeftijdscategorieen'.

VALUE LABELS SD_Lft_S_Cat4

1 '≤24'

2 '25-29'

3 '30-34'

4 '≥35'

999 'missing'.

FREQUENCIES SD_Lft_S_Cat4.

NUMERIC SD_Lft_M_Cat4 (F2.0).

COMPUTE SD_Lft_M_Cat4 =SD_Lft_S_Cat4.

VARIABLE LABELS SD_Lft_M_Cat4 'leeftijdscategorieen - Missing'.

VALUE LABELS SD_Lft_M_Cat4

1 '≤24'

2 '25-29'

3 '30-34'

4 '≥35'

999 'missing'.

MISSING VALUES SD_Lft_M_Cat4 (999).

FREQUENCIES SD_Lft_M_Cat4.

*PARITEIT .

NUMERIC EE_Par_S_Cat2 (F2.0).

COMPUTE EE_Par_S_Cat2 = -999.

IF (EE_Par_S_Cat3 = 1) EE_Par_S_Cat2 = 1.

IF (EE_Par_S_Cat3 = 2 OR EE_Par_S_Cat3 = 3 ) EE_Par_S_Cat2 = 2.

IF (EE_Par_S_Cat3 = 999) EE_Par_S_Cat2 = 999.

VARIABLE LABELS EE_Par_S_Cat2 'nulli vs primi/multipara'.

VALUE LABELS EE_Par_S_Cat2

1 'nulli para'

2 'primi/multipara'

999 'missing'.

FREQUENCIES EE_Par_S_Cat2.

NUMERIC EE_Par_M_Cat2 (F2.0).

COMPUTE EE_Par_M_Cat2 = EE_Par_S_Cat2.

VARIABLE LABELS EE_Par_M_Cat2 'nulli vs primi/multipara - Missing'.

VALUE LABELS EE_Par_M_Cat2

1 'nulli para'

2 'primi/multipara'

999 'missing'.

MISSING VALUES EE_Par_M_Cat2 (999).

FREQUENCIES EE_Par_M_Cat2.

*Picker - vraag.

NUMERIC OV_All_S_Cat5 (F2.0).

COMPUTE OV_All_S_Cat5 =-999.

IF (OV_All_S=1 OR OV_All_S=2 OR OV_All_S=3 OR OV_All_S=4 OR OV_All_S=5 OR OV_All_S=6) OV_All_S_Cat5 =1.

IF (OV_All_S=7) OV_All_S_Cat5 =2.

IF (OV_All_S=8) OV_All_S_Cat5 =3.

IF (OV_All_S=9) OV_All_S_Cat5 =4.

IF (OV_All_S=10) OV_All_S_Cat5 =5.

IF (OV_All_S=999) OV_All_S_Cat5 =999.

VARIABLE LABELS OV_All_S_Cat5 'overall cijfer - SYSMIS'.

VALUE LABELS OV_All_S_Cat5

1 '≤6'

2 '7'

3 '8'

4 '9'

5 '10'

999 'missing'.

FREQUENCIES OV_All_S_Cat5.

NUMERIC OV_All_M_Cat5 (F2.0).

COMPUTE OV_All_M_Cat5 =-999.

IF (OV_All_S=1 OR OV_All_S=2 OR OV_All_S=3 OR OV_All_S=4 OR OV_All_S=5 OR OV_All_S=6) OV_All_M_Cat5 =1.

IF (OV_All_S=7) OV_All_M_Cat5 =2.

IF (OV_All_S=8) OV_All_M_Cat5 =3.

IF (OV_All_S=9) OV_All_M_Cat5 =4.

IF (OV_All_S=10) OV_All_M_Cat5 =5.

IF (OV_All_S=999) OV_All_M_Cat5 =999.

VARIABLE LABELS OV_All_M_Cat5 'overall cijfer - Missing'.

VALUE LABELS OV_All_M_Cat5

1 '≤6'

2 '7'

3 '8'

4 '9'

5 '10'

999 'missing'.

MISSING VALUES OV_All_M_Cat5 (999).

FREQUENCIES OV_All_M_Cat5.

*ZORGPROCES .

*afgewisseld opgnomen in groep 1e naar 2e lijn verwezen.

*iemand 2e lijn gestart, verwezen 1e lijn en daarna 2e lijn = 2 lijn.

*groepen antenataal.

NUMERIC ZZ_Pro_S_Cat6 (F2.0).

COMPUTE ZZ_Pro_S_Cat6=-999.

IF (ZZ_Con_S=1 AND ZZ_Cvk_S=1) ZZ_Pro_S_Cat6=1.

IF (ZZ_Con_S=1 AND ZZ_Cvk_S=4) ZZ_Pro_S_Cat6=1.

IF (ZZ_Con_S=3 AND (ZZ_Cha_S=1 OR ZZ_Cha_S=2)) ZZ_Pro_S_Cat6=1.

IF (ZZ_Con_S=4 AND (ZZ_Mcb_S=1 OR ZZ_Mcb_S=3) AND (ZZ_Mce_S=1 OR ZZ_Mce_S=3)) ZZ_Pro_S_Cat6=1.

IF (ZZ_Con_S=1 AND ZZ_Cvk_S=2) ZZ_Pro_S_Cat6=2.

IF (ZZ_Con_S=3 AND ZZ_Cha_S=3) ZZ_Pro_S_Cat6=2.

IF (ZZ_Con_S=1 AND ZZ_Cvk_S=3) ZZ_Pro_S_Cat6=3.

IF (ZZ_Con_S=3 AND ZZ_Cha_S=4) ZZ_Pro_S_Cat6=3.

IF (ZZ_Con_S=4 AND (ZZ_Mcb_S=1 OR ZZ_Mcb_S=3) AND ZZ_Mce_S=2) ZZ_Pro_S_Cat6=3.

IF (ZZ_Con_S=1 AND ZZ_Cvk_S=4) ZZ_Pro_S_Cat6=6.

IF (ZZ_Con_S=2 AND ZZ_Cgy_S=4) ZZ_Pro_S_Cat6=6.

IF (ZZ_Con_S=4 AND (ZZ_Mcb_S=4 OR ZZ_Mcb_S=5 OR ZZ_Mce_S=4 OR ZZ_Mce_S=5)) ZZ_Pro_S_Cat6=6.

IF (ZZ_Con_S=2 AND ZZ_Cgy_S=3) ZZ_Pro_S_Cat6=4.

IF (ZZ_Con_S=4 AND ZZ_Mcb_S=2 AND (ZZ_Mce_S=1 OR ZZ_Mce_S=3)) ZZ_Pro_S_Cat6=4.

IF (ZZ_Con_S=2 AND ZZ_Cgy_S=1) ZZ_Pro_S_Cat6=5.

IF (ZZ_Con_S=2 AND ZZ_Cgy_S=2) ZZ_Pro_S_Cat6=5.

IF (ZZ_Con_S=4 AND ZZ_Mcb_S=2 AND ZZ_Mce_S=2) ZZ_Pro_S_Cat6=5.

IF (ZZ_Con_S=999) ZZ_Pro_S_Cat6=999.

IF (ZZ_Con_S =1 AND ZZ_Cvk_S =999) ZZ_Pro_S_Cat6= 999.

IF (ZZ_Con_S=2 AND ZZ_Cgy_S=999) ZZ_Pro_S_Cat6=999.

IF (ZZ_Con_S=3 AND ZZ_Cha_S=999) ZZ_Pro_S_Cat6=999.

IF (ZZ_Con_S=4 AND (ZZ_Mcb_S=999 OR ZZ_Mce_S=999)) ZZ_Pro_S_Cat6=999.

VARIABLE LABELS ZZ_Pro_S_Cat6 'Zorgproces tijdens zwangerschap'.

VALUE LABELS ZZ_Pro_S_Cat6

1 'bij verloskundige/huisarts begonnen en geeindigd'

2 'bij verloskundige/huisarts begonnen, tijdelijk verwezen naar gynaecoloog'

3 'bij verloskundige/huisarts begonnen, permantent verwezen naar gynaecoloog'

4 'bij gynaecoloog begonnen, permantent verwezen naar verloskundige'

5 'bij gynaecoloog begonnen en geeindigd'

6 'integrale zorg'

999 'missing'.

FREQUENCIES ZZ_Pro_S_Cat6.

NUMERIC ZB_Pro_S_Cat5 (F2.0).

COMPUTE ZB_Pro_S_Cat5 = -999.

IF (ZB_Sbb_S_Cat4 = 1 AND ZB_Seb_S_Cat4 = 1) ZB_Pro_S_Cat5 =1.

IF (ZB_Sbb_S_Cat4 = 4 AND ZB_Seb_S_Cat4 = 1) ZB_Pro_S_Cat5 =1.

IF ((ZB_Sbb_S_Cat4=1 OR ZB_Sbb_S_Cat4 = 2) AND ZB_Seb_S_Cat4 = 2) ZB_Pro_S_Cat5 =2.

IF ((ZB_Sbb_S_Cat4=4) AND ZB_Seb_S_Cat4 = 2) ZB_Pro_S_Cat5 =2.

IF ((ZB_Sbb_S_Cat4=1 OR ZB_Sbb_S_Cat4=2 OR ZB_Sbb_S_Cat4 = 3) AND ZB_Seb_S_Cat4 = 3) ZB_Pro_S_Cat5 =3.

IF ((ZB_Sbb_S_Cat4=1 OR ZB_Sbb_S_Cat4=2 OR ZB_Sbb_S_Cat4=3) AND ZB_Seb_S_Cat4 = 4) ZB_Pro_S_Cat5 =4.

IF (ZB_Sbb_S_Cat4 = 4 AND ZB_Seb_S_Cat4 = 4) ZB_Pro_S_Cat5 =5.

IF (ZB_Sbb_S_Cat4 = 4 AND ZB_Seb_S_Cat4 = 3) ZB_Pro_S_Cat5 =5.

IF (ZB_Sbb_S_Cat4 = 999 OR ZB_Seb_S_Cat4 = 999) ZB_Pro_S_Cat5 =999.

VARIABLE LABELS ZB_Pro_S_Cat5 'zorgproces tijdens de bevalling'.

VALUE LABELS ZB_Pro_S_Cat5

1 'thuis, onder leiding van 1e lijns verloskundige'

2 'geboortecentrum, onder leiding van 1e lijnsverloskundige'

3 'poliklinisch, onder leiding van 1e lijnsverloskundige'

4 'gestart onder leiding van 1e lijns verloskundige, verwezen naar ziekenhuis tijdens bevalling'

5 'ziekenhuis, onderleiding van 2e lijns verloskundige'

999 'missing'.

FREQUENCIES ZB_Pro_S_Cat5 .

NUMERIC Zorgproces_S_Cat4 (F2.0).

COMPUTE Zorgproces_S_Cat4 = -999.

IF (ZZ_Pro_S_Cat6 = 1 AND (ZB_Pro_S_Cat5 =1 OR ZB_Pro_S_Cat5=2 OR ZB_Pro_S_Cat5=3)) Zorgproces_S_Cat4 = 1.

IF (ZZ_Pro_S_Cat6 = 2 AND (ZB_Pro_S_Cat5 =1 OR ZB_Pro_S_Cat5=2 OR ZB_Pro_S_Cat5=3)) Zorgproces_S_Cat4 = 1.

IF (ZZ_Pro_S_Cat6 = 4 AND (ZB_Pro_S_Cat5 =1 OR ZB_Pro_S_Cat5=2 OR ZB_Pro_S_Cat5=3)) Zorgproces_S_Cat4 = 1.

IF (ZZ_Pro_S_Cat6 = 6 AND (ZB_Pro_S_Cat5 =1 OR ZB_Pro_S_Cat5=2 OR ZB_Pro_S_Cat5=3)) Zorgproces_S_Cat4 = 1.

IF (ZZ_Pro_S_Cat6 = 3 AND ZB_Pro_S_Cat5 =1 ) Zorgproces_S_Cat4 = 1.

IF (ZZ_Pro_S_Cat6 = 5 AND (ZB_Pro_S_Cat5 =5)) Zorgproces_S_Cat4 = 2.

IF (ZZ_Pro_S_Cat6 = 5 AND (ZB_Pro_S_Cat5 =4)) Zorgproces_S_Cat4 = 2.

IF (ZZ_Pro_S_Cat6 = 3 AND (ZB_Pro_S_Cat5 =4)) Zorgproces_S_Cat4 = 2.

IF (ZZ_Pro_S_Cat6 = 6 AND (ZB_Pro_S_Cat5 =5)) Zorgproces_S_Cat4 = 2.

IF (ZZ_Pro_S_Cat6 = 5 AND (ZB_Pro_S_Cat5 =1 OR ZB_Pro_S_Cat5=2 OR ZB_Pro_S_Cat5=3)) Zorgproces_S_Cat4 = 2.

IF (ZZ_Pro_S_Cat6 = 3 AND (ZB_Pro_S_Cat5 =5)) Zorgproces_S_Cat4 = 3.

IF (ZZ_Pro_S_Cat6 = 3 AND (ZB_Pro_S_Cat5 =3)) Zorgproces_S_Cat4 = 3.

IF (ZZ_Pro_S_Cat6 = 1 AND (ZB_Pro_S_Cat5 =4)) Zorgproces_S_Cat4 = 4.

IF (ZZ_Pro_S_Cat6 = 2 AND (ZB_Pro_S_Cat5 =4)) Zorgproces_S_Cat4 = 4.

IF (ZZ_Pro_S_Cat6 = 4 AND (ZB_Pro_S_Cat5 =4)) Zorgproces_S_Cat4 = 4.

IF (ZZ_Pro_S_Cat6 = 4 AND (ZB_Pro_S_Cat5 =5)) Zorgproces_S_Cat4 = 4.

IF (ZZ_Pro_S_Cat6 = 2 AND (ZB_Pro_S_Cat5 =5)) Zorgproces_S_Cat4 = 4.

IF (ZZ_Pro_S_Cat6 = 1 AND (ZB_Pro_S_Cat5 =5)) Zorgproces_S_Cat4 = 4.

IF (ZZ_Pro_S_Cat6 = 6 AND (ZB_Pro_S_Cat5 =4)) Zorgproces_S_Cat4 = 4.

IF (ZZ_Pro_S_Cat6 = 999 OR (ZB_Pro_S_Cat5 =999)) Zorgproces_S_Cat4 = 999.

VARIABLE LABELS Zorgproces_S_Cat4 'zorgproces tijdens zwangerschap en bevalling'.

VALUE LABELS Zorgproces_S_Cat4

1 'volledig eerste lijn'

2 'volledig tweede lijn'

3 'permanent verwezen naar tweede lijn tijdens zwangerschap'

4 'tijdens bevalling verwezen naar tweede lijn'

999 'missing'.

FREQUENCIES Zorgproces_S_Cat4.

NUMERIC Zorgproces_M_Cat4 (F2.0).

COMPUTE Zorgproces_M_Cat4 = Zorgproces_S_Cat4.

VARIABLE LABELS Zorgproces_M_Cat4 'zorgproces tijdens zwangerschap en bevalling - Missing'.

VALUE LABELS Zorgproces_M_Cat4

1 'volledig eerste lijn'

2 'volledig tweede lijn'

3 'permanent verwezen naar tweede lijn tijdens zwangerschap'

4 'tijdens bevalling verwezen naar tweede lijn'

999 'missing'.

MISSING VALUES Zorgproces_M_Cat4 (999).

FREQUENCIES Zorgproces_M_Cat4.

* Keizersnee tijdens de bevalling.

NUMERIC ZB_Kjn_S_Cat3 (F2.0).

COMPUTE ZB_Kjn_S_Cat3 = -999.

IF (ZB_Mbv_S=1 OR ZB_Mbv_S=2 OR ZB_Mbv_S= 3 OR ZB_Mbv_S=4) ZB_Kjn_S_Cat3 = 1.

IF (ZB_Mbv_S=5) ZB_Kjn_S_Cat3 = 2.

IF (ZB_Mbv_S=6) ZB_Kjn_S_Cat3 = 3.

IF (ZB_Mbv_S=999) ZB_Kjn_S_Cat3 = 999.

VARIABLE LABELS ZB_Kjn_S_Cat3 'keizersnee tijdens de bevalling, cat 3 - Sysmis'.

VALUE LABELS ZB_Kjn_S_Cat3

1 'no'

2 'geplande keizersnee'

3 'spoed keizersnee'

999 'missing'.

FREQUENCIES ZB_Kjn_S_Cat3.

NUMERIC ZB_Kjn_M_Cat3 (F2.0).

COMPUTE ZB_Kjn_M_Cat3 = -999.

IF (ZB_Mbv_S=1 OR ZB_Mbv_S=2 OR ZB_Mbv_S= 3 OR ZB_Mbv_S=4) ZB_Kjn_M_Cat3 = 1.

IF (ZB_Mbv_S=5) ZB_Kjn_M_Cat3 = 2.

IF (ZB_Mbv_S=6) ZB_Kjn_M_Cat3 = 3.

IF (ZB_Mbv_S=999) ZB_Kjn_M_Cat3 = 999.

VARIABLE LABELS ZB_Kjn_M_Cat3 'keizersnee tijdens de bevalling, cat 3 - Missing'.

VALUE LABELS ZB_Kjn_M_Cat3

1 'no'

2 'geplande keizersnee'

3 'spoed keizersnee'

999 'missing'.

MISSING VALUES ZB_Kjn_M_Cat3 (999).

FREQUENCIES ZB_Kjn_M_Cat3.

NUMERIC ZB_Kjn_M_Cat2 (F2.0).

COMPUTE ZB_Kjn_M_Cat2 = -999.

IF (ZB_Mbv_S=1 OR ZB_Mbv_S=2 OR ZB_Mbv_S =3 OR ZB_Mbv_S=4) ZB_Kjn_M_Cat2 = 1.

IF (ZB_Mbv_S=5) ZB_Kjn_M_Cat2 = 2.

IF (ZB_Mbv_S=6) ZB_Kjn_M_Cat2 = 3.

IF (ZB_Mbv_S=999) ZB_Kjn_M_Cat2 = 999.

VARIABLE LABELS ZB_Kjn_M_Cat2 'keizersnee tijdens de bevalling, cat 2 - Missing'.

VALUE LABELS ZB_Kjn_M_Cat2

1 'no'

2 'geplande keizersnee'

3 'spoed keizersnee'

999 'missing'.

MISSING VALUES ZB_Kjn_M_Cat2 (1 999).

FREQUENCIES ZB_Kjn_M_Cat2.

*KENNEN ZORGVERLENER.

NUMERIC OV_Kza_S_Cat2 (F2.0).

COMPUTE OV_Kza_S_Cat2 = -999.

IF (OV_Kza_S = 1 OR OV_Kza_S=2) OV_Kza_S_Cat2 = 1.

IF (OV_Kza_S = 3 OR OV_Kza_S=4) OV_Kza_S_Cat2 = 2.

IF (OV_Kza_S = 999) OV_Kza_S_Cat2 = 999.

VARIABLE LABELS OV_Kza_S_Cat2 'Kennen zorgverlener leiding bevalling - Cat 2'.

VALUE LABELS OV_Kza_S_Cat2

1 'client kent zorgverlener die leiding had over bevalling'

2 'client kent zorgverlener die leiding had over bevalling NIET'

999 'missing'.

FREQUENCIES OV_Kza_S_Cat2.

NUMERIC OV_Kza_M_Cat2 (F2.0).

COMPUTE OV_Kza_M_Cat2 = OV_Kza_S_Cat2.

VARIABLE LABELS OV_Kza_M_Cat2 'Kennen zorgverlener leiding bevalling - Cat 2, Missing'.

VALUE LABELS OV_Kza_M_Cat2

1 'client kent zorgverlener die leiding had over bevalling'

2 'client kent zorgverlener die leiding had over bevalling NIET'

999 'missing'.

MISSING VALUES OV_Kza_M_Cat2 (999).

FREQUENCIES OV_Kza_M_Cat2.

*KANTOORTIJD.

NUMERIC ZB_Kan_S_Cat2 (F2.0).

COMPUTE ZB_Kan_S_Cat2 = -999.

IF (ZB_Tbv_S=1 AND ZB_Dag_S=1) ZB_Kan_S_Cat2 = 1.

IF (ZB_Tbv_S=2 OR (ZB_Dag_S=2 OR ZB_Dag_S=3)) ZB_Kan_S_Cat2 = 2.

IF (ZB_Tbv_S=999 OR ZB_Dag_S = 999) ZB_Kan_S_Cat2 = 999.

VARIABLE LABELS ZB_Kan_S_Cat2 'Bevallen kantoor tijd - Cat2'.

VALUE LABELS ZB_Kan_S_Cat2

1 "Tussen 8:00-17:00, op doordeweekse dag"

2 "Tussen 17:00-8:00 en/of dag in het weekend"

999 'missing'.

FREQUENCIES ZB_Kan_S_Cat2.

NUMERIC ZB_Kan_M_Cat2 (F2.0).

COMPUTE ZB_Kan_M_Cat2 = ZB_Kan_S_Cat2.

VARIABLE LABELS ZB_Kan_M_Cat2 'Bevallen kantoor tijd - Cat2, Missing'.

VALUE LABELS ZB_Kan_M_Cat2

1 "Tussen 8:00-17:00, op doordeweekse dag"

2 "Tussen 17:00-8:00 en/of dag in het weekend"

999 'missing'.

MISSING VALUES ZB_Kan_M_Cat2 (999).

FREQUENCIES ZB_Kan_M_Cat2.

* AANTAL ZIEKENHUIZEN PER STAD.

NUMERIC ZB_Urb_S (F2.0).

COMPUTE ZB_Urb_S = 3.

IF ( SD_Pst = 1011 ) ZB_Urb_S = 1 .

IF ( SD_Pst = 1012 ) ZB_Urb_S = 1 .

IF ( SD_Pst = 1013 ) ZB_Urb_S = 1 .

IF ( SD_Pst = 1014 ) ZB_Urb_S = 1 .

IF ( SD_Pst = 1015 ) ZB_Urb_S = 1 .

IF ( SD_Pst = 1016 ) ZB_Urb_S = 1 .

IF ( SD_Pst = 1017 ) ZB_Urb_S = 1 .

IF ( SD_Pst = 1018 ) ZB_Urb_S = 1 .

IF ( SD_Pst = 1019 ) ZB_Urb_S = 1 .

IF ( SD_Pst = 1021 ) ZB_Urb_S = 1 .

IF ( SD_Pst = 1022 ) ZB_Urb_S = 1 .

IF ( SD_Pst = 1023 ) ZB_Urb_S = 1 .

IF ( SD_Pst = 1024 ) ZB_Urb_S = 1 .

IF ( SD_Pst = 1025 ) ZB_Urb_S = 1 .

IF ( SD_Pst = 1026 ) ZB_Urb_S = 1 .

IF ( SD_Pst = 1027 ) ZB_Urb_S = 1 .

IF ( SD_Pst = 1028 ) ZB_Urb_S = 1 .

IF ( SD_Pst = 1031 ) ZB_Urb_S = 1 .

IF ( SD_Pst = 1032 ) ZB_Urb_S = 1 .

IF ( SD_Pst = 1033 ) ZB_Urb_S = 1 .

IF ( SD_Pst = 1034 ) ZB_Urb_S = 1 .

IF ( SD_Pst = 1035 ) ZB_Urb_S = 1 .

IF ( SD_Pst = 1036 ) ZB_Urb_S = 1 .

IF ( SD_Pst = 1037 ) ZB_Urb_S = 1 .

IF ( SD_Pst = 1041 ) ZB_Urb_S = 1 .

IF ( SD_Pst = 1043 ) ZB_Urb_S = 1 .

IF ( SD_Pst = 1044 ) ZB_Urb_S = 1 .

IF ( SD_Pst = 1045 ) ZB_Urb_S = 1 .

IF ( SD_Pst = 1046 ) ZB_Urb_S = 1 .

IF ( SD_Pst = 1047 ) ZB_Urb_S = 1 .

IF ( SD_Pst = 1051 ) ZB_Urb_S = 1 .

IF ( SD_Pst = 1052 ) ZB_Urb_S = 1 .

IF ( SD_Pst = 1053 ) ZB_Urb_S = 1 .

IF ( SD_Pst = 1054 ) ZB_Urb_S = 1 .

IF ( SD_Pst = 1055 ) ZB_Urb_S = 1 .

IF ( SD_Pst = 1056 ) ZB_Urb_S = 1 .

IF ( SD_Pst = 1057 ) ZB_Urb_S = 1 .

IF ( SD_Pst = 1058 ) ZB_Urb_S = 1 .

IF ( SD_Pst = 1059 ) ZB_Urb_S = 1 .

IF ( SD_Pst = 1060 ) ZB_Urb_S = 1 .

IF ( SD_Pst = 1061 ) ZB_Urb_S = 1 .

IF ( SD_Pst = 1062 ) ZB_Urb_S = 1 .

IF ( SD_Pst = 1063 ) ZB_Urb_S = 1 .

IF ( SD_Pst = 1064 ) ZB_Urb_S = 1 .

IF ( SD_Pst = 1065 ) ZB_Urb_S = 1 .

IF ( SD_Pst = 1066 ) ZB_Urb_S = 1 .

IF ( SD_Pst = 1067 ) ZB_Urb_S = 1 .

IF ( SD_Pst = 1068 ) ZB_Urb_S = 1 .

IF ( SD_Pst = 1069 ) ZB_Urb_S = 1 .

IF ( SD_Pst = 1071 ) ZB_Urb_S = 1 .

IF ( SD_Pst = 1072 ) ZB_Urb_S = 1 .

IF ( SD_Pst = 1073 ) ZB_Urb_S = 1 .

IF ( SD_Pst = 1074 ) ZB_Urb_S = 1 .

IF ( SD_Pst = 1075 ) ZB_Urb_S = 1 .

IF ( SD_Pst = 1076 ) ZB_Urb_S = 1 .

IF ( SD_Pst = 1077 ) ZB_Urb_S = 1 .

IF ( SD_Pst = 1078 ) ZB_Urb_S = 1 .

IF ( SD_Pst = 1079 ) ZB_Urb_S = 1 .

IF ( SD_Pst = 1081 ) ZB_Urb_S = 1 .

IF ( SD_Pst = 1082 ) ZB_Urb_S = 1 .

IF ( SD_Pst = 1083 ) ZB_Urb_S = 1 .

IF ( SD_Pst = 1086 ) ZB_Urb_S = 1 .

IF ( SD_Pst = 1087 ) ZB_Urb_S = 1 .

IF ( SD_Pst = 1091 ) ZB_Urb_S = 1 .

IF ( SD_Pst = 1092 ) ZB_Urb_S = 1 .

IF ( SD_Pst = 1093 ) ZB_Urb_S = 1 .

IF ( SD_Pst = 1094 ) ZB_Urb_S = 1 .

IF ( SD_Pst = 1095 ) ZB_Urb_S = 1 .

IF ( SD_Pst = 1096 ) ZB_Urb_S = 1 .

IF ( SD_Pst = 1097 ) ZB_Urb_S = 1 .

IF ( SD_Pst = 1098 ) ZB_Urb_S = 1 .

IF ( SD_Pst = 1099 ) ZB_Urb_S = 1 .

IF ( SD_Pst = 1101 ) ZB_Urb_S = 1 .

IF ( SD_Pst = 1102 ) ZB_Urb_S = 1 .

IF ( SD_Pst = 1103 ) ZB_Urb_S = 1 .

IF ( SD_Pst = 1104 ) ZB_Urb_S = 1 .

IF ( SD_Pst = 1105 ) ZB_Urb_S = 1 .

IF ( SD_Pst = 1106 ) ZB_Urb_S = 1 .

IF ( SD_Pst = 1107 ) ZB_Urb_S = 1 .

IF ( SD_Pst = 1108 ) ZB_Urb_S = 1 .

IF ( SD_Pst = 1109 ) ZB_Urb_S = 1 .

IF ( SD_Pst = 2491 ) ZB_Urb_S = 1 .

IF ( SD_Pst = 2491 ) ZB_Urb_S = 1 .

IF ( SD_Pst = 2493 ) ZB_Urb_S = 1 .

IF ( SD_Pst = 2495 ) ZB_Urb_S = 1 .

IF ( SD_Pst = 2496 ) ZB_Urb_S = 1 .

IF ( SD_Pst = 2497 ) ZB_Urb_S = 1 .

IF ( SD_Pst = 2498 ) ZB_Urb_S = 1 .

IF ( SD_Pst = 2511 ) ZB_Urb_S = 1 .

IF ( SD_Pst = 2512 ) ZB_Urb_S = 1 .

IF ( SD_Pst = 2513 ) ZB_Urb_S = 1 .

IF ( SD_Pst = 2514 ) ZB_Urb_S = 1 .

IF ( SD_Pst = 2515 ) ZB_Urb_S = 1 .

IF ( SD_Pst = 2516 ) ZB_Urb_S = 1 .

IF ( SD_Pst = 2517 ) ZB_Urb_S = 1 .

IF ( SD_Pst = 2518 ) ZB_Urb_S = 1 .

IF ( SD_Pst = 2521 ) ZB_Urb_S = 1 .

IF ( SD_Pst = 2522 ) ZB_Urb_S = 1 .

IF ( SD_Pst = 2523 ) ZB_Urb_S = 1 .

IF ( SD_Pst = 2524 ) ZB_Urb_S = 1 .

IF ( SD_Pst = 2525 ) ZB_Urb_S = 1 .

IF ( SD_Pst = 2526 ) ZB_Urb_S = 1 .

IF ( SD_Pst = 2531 ) ZB_Urb_S = 1 .

IF ( SD_Pst = 2532 ) ZB_Urb_S = 1 .

IF ( SD_Pst = 2533 ) ZB_Urb_S = 1 .

IF ( SD_Pst = 2541 ) ZB_Urb_S = 1 .

IF ( SD_Pst = 2542 ) ZB_Urb_S = 1 .

IF ( SD_Pst = 2543 ) ZB_Urb_S = 1 .

IF ( SD_Pst = 2544 ) ZB_Urb_S = 1 .

IF ( SD_Pst = 2545 ) ZB_Urb_S = 1 .

IF ( SD_Pst = 2546 ) ZB_Urb_S = 1 .

IF ( SD_Pst = 2547 ) ZB_Urb_S = 1 .

IF ( SD_Pst = 2548 ) ZB_Urb_S = 1 .

IF ( SD_Pst = 2551 ) ZB_Urb_S = 1 .

IF ( SD_Pst = 2552 ) ZB_Urb_S = 1 .

IF ( SD_Pst = 2553 ) ZB_Urb_S = 1 .

IF ( SD_Pst = 2554 ) ZB_Urb_S = 1 .

IF ( SD_Pst = 2555 ) ZB_Urb_S = 1 .

IF ( SD_Pst = 2561 ) ZB_Urb_S = 1 .

IF ( SD_Pst = 2562 ) ZB_Urb_S = 1 .

IF ( SD_Pst = 2563 ) ZB_Urb_S = 1 .

IF ( SD_Pst = 2564 ) ZB_Urb_S = 1 .

IF ( SD_Pst = 2565 ) ZB_Urb_S = 1 .

IF ( SD_Pst = 2566 ) ZB_Urb_S = 1 .

IF ( SD_Pst = 2571 ) ZB_Urb_S = 1 .

IF ( SD_Pst = 2572 ) ZB_Urb_S = 1 .

IF ( SD_Pst = 2573 ) ZB_Urb_S = 1 .

IF ( SD_Pst = 2574 ) ZB_Urb_S = 1 .

IF ( SD_Pst = 2581 ) ZB_Urb_S = 1 .

IF ( SD_Pst = 2582 ) ZB_Urb_S = 1 .

IF ( SD_Pst = 2583 ) ZB_Urb_S = 1 .

IF ( SD_Pst = 2584 ) ZB_Urb_S = 1 .

IF ( SD_Pst = 2585 ) ZB_Urb_S = 1 .

IF ( SD_Pst = 2586 ) ZB_Urb_S = 1 .

IF ( SD_Pst = 2587 ) ZB_Urb_S = 1 .

IF ( SD_Pst = 2591 ) ZB_Urb_S = 1 .

IF ( SD_Pst = 2592 ) ZB_Urb_S = 1 .

IF ( SD_Pst = 2593 ) ZB_Urb_S = 1 .

IF ( SD_Pst = 2594 ) ZB_Urb_S = 1 .

IF ( SD_Pst = 2595 ) ZB_Urb_S = 1 .

IF ( SD_Pst = 2596 ) ZB_Urb_S = 1 .

IF ( SD_Pst = 2597 ) ZB_Urb_S = 1 .

IF ( SD_Pst = 3011 ) ZB_Urb_S = 1 .

IF ( SD_Pst = 3012 ) ZB_Urb_S = 1 .

IF ( SD_Pst = 3013 ) ZB_Urb_S = 1 .

IF ( SD_Pst = 3014 ) ZB_Urb_S = 1 .

IF ( SD_Pst = 3015 ) ZB_Urb_S = 1 .

IF ( SD_Pst = 3016 ) ZB_Urb_S = 1 .

IF ( SD_Pst = 3021 ) ZB_Urb_S = 1 .

IF ( SD_Pst = 3022 ) ZB_Urb_S = 1 .

IF ( SD_Pst = 3023 ) ZB_Urb_S = 1 .

IF ( SD_Pst = 3024 ) ZB_Urb_S = 1 .

IF ( SD_Pst = 3025 ) ZB_Urb_S = 1 .

IF ( SD_Pst = 3026 ) ZB_Urb_S = 1 .

IF ( SD_Pst = 3027 ) ZB_Urb_S = 1 .

IF ( SD_Pst = 3028 ) ZB_Urb_S = 1 .

IF ( SD_Pst = 3029 ) ZB_Urb_S = 1 .

IF ( SD_Pst = 3031 ) ZB_Urb_S = 1 .

IF ( SD_Pst = 3032 ) ZB_Urb_S = 1 .

IF ( SD_Pst = 3033 ) ZB_Urb_S = 1 .

IF ( SD_Pst = 3034 ) ZB_Urb_S = 1 .

IF ( SD_Pst = 3035 ) ZB_Urb_S = 1 .

IF ( SD_Pst = 3036 ) ZB_Urb_S = 1 .

IF ( SD_Pst = 3037 ) ZB_Urb_S = 1 .

IF ( SD_Pst = 3038 ) ZB_Urb_S = 1 .

IF ( SD_Pst = 3039 ) ZB_Urb_S = 1 .

IF ( SD_Pst = 3041 ) ZB_Urb_S = 1 .

IF ( SD_Pst = 3042 ) ZB_Urb_S = 1 .

IF ( SD_Pst = 3043 ) ZB_Urb_S = 1 .

IF ( SD_Pst = 3044 ) ZB_Urb_S = 1 .

IF ( SD_Pst = 3045 ) ZB_Urb_S = 1 .

IF ( SD_Pst = 3046 ) ZB_Urb_S = 1 .

IF ( SD_Pst = 3047 ) ZB_Urb_S = 1 .

IF ( SD_Pst = 3051 ) ZB_Urb_S = 1 .

IF ( SD_Pst = 3052 ) ZB_Urb_S = 1 .

IF ( SD_Pst = 3053 ) ZB_Urb_S = 1 .

IF ( SD_Pst = 3054 ) ZB_Urb_S = 1 .

IF ( SD_Pst = 3055 ) ZB_Urb_S = 1 .

IF ( SD_Pst = 3056 ) ZB_Urb_S = 1 .

IF ( SD_Pst = 3059 ) ZB_Urb_S = 1 .

IF ( SD_Pst = 3061 ) ZB_Urb_S = 1 .

IF ( SD_Pst = 3062 ) ZB_Urb_S = 1 .

IF ( SD_Pst = 3063 ) ZB_Urb_S = 1 .

IF ( SD_Pst = 3064 ) ZB_Urb_S = 1 .

IF ( SD_Pst = 3065 ) ZB_Urb_S = 1 .

IF ( SD_Pst = 3066 ) ZB_Urb_S = 1 .

IF ( SD_Pst = 3067 ) ZB_Urb_S = 1 .

IF ( SD_Pst = 3068 ) ZB_Urb_S = 1 .

IF ( SD_Pst = 3069 ) ZB_Urb_S = 1 .

IF ( SD_Pst = 3071 ) ZB_Urb_S = 1 .

IF ( SD_Pst = 3072 ) ZB_Urb_S = 1 .

IF ( SD_Pst = 3073 ) ZB_Urb_S = 1 .

IF ( SD_Pst = 3074 ) ZB_Urb_S = 1 .

IF ( SD_Pst = 3075 ) ZB_Urb_S = 1 .

IF ( SD_Pst = 3076 ) ZB_Urb_S = 1 .

IF ( SD_Pst = 3077 ) ZB_Urb_S = 1 .

IF ( SD_Pst = 3078 ) ZB_Urb_S = 1 .

IF ( SD_Pst = 3079 ) ZB_Urb_S = 1 .

IF ( SD_Pst = 3081 ) ZB_Urb_S = 1 .

IF ( SD_Pst = 3082 ) ZB_Urb_S = 1 .

IF ( SD_Pst = 3083 ) ZB_Urb_S = 1 .

IF ( SD_Pst = 3084 ) ZB_Urb_S = 1 .

IF ( SD_Pst = 3085 ) ZB_Urb_S = 1 .

IF ( SD_Pst = 3086 ) ZB_Urb_S = 1 .

IF ( SD_Pst = 3087 ) ZB_Urb_S = 1 .

IF ( SD_Pst = 3088 ) ZB_Urb_S = 1 .

IF ( SD_Pst = 3089 ) ZB_Urb_S = 1 .

IF ( SD_Pst = 3151 ) ZB_Urb_S = 1 .

IF ( SD_Pst = 3191 ) ZB_Urb_S = 1 .

IF ( SD_Pst = 3192 ) ZB_Urb_S = 1 .

IF ( SD_Pst = 3193 ) ZB_Urb_S = 1 .

IF ( SD_Pst = 3194 ) ZB_Urb_S = 1 .

IF ( SD_Pst = 3195 ) ZB_Urb_S = 1 .

IF ( SD_Pst = 3197 ) ZB_Urb_S = 1 .

IF ( SD_Pst = 3451 ) ZB_Urb_S = 1 .

IF ( SD_Pst = 3452 ) ZB_Urb_S = 1 .

IF ( SD_Pst = 3453 ) ZB_Urb_S = 1 .

IF ( SD_Pst = 3454 ) ZB_Urb_S = 1 .

IF ( SD_Pst = 3455 ) ZB_Urb_S = 1 .

IF ( SD_Pst = 3511 ) ZB_Urb_S = 1 .

IF ( SD_Pst = 3512 ) ZB_Urb_S = 1 .

IF ( SD_Pst = 3513 ) ZB_Urb_S = 1 .

IF ( SD_Pst = 3514 ) ZB_Urb_S = 1 .

IF ( SD_Pst = 3515 ) ZB_Urb_S = 1 .

IF ( SD_Pst = 3521 ) ZB_Urb_S = 1 .

IF ( SD_Pst = 3522 ) ZB_Urb_S = 1 .

IF ( SD_Pst = 3523 ) ZB_Urb_S = 1 .

IF ( SD_Pst = 3524 ) ZB_Urb_S = 1 .

IF ( SD_Pst = 3525 ) ZB_Urb_S = 1 .

IF ( SD_Pst = 3526 ) ZB_Urb_S = 1 .

IF ( SD_Pst = 3527 ) ZB_Urb_S = 1 .

IF ( SD_Pst = 3528 ) ZB_Urb_S = 1 .

IF ( SD_Pst = 3531 ) ZB_Urb_S = 1 .

IF ( SD_Pst = 3532 ) ZB_Urb_S = 1 .

IF ( SD_Pst = 3533 ) ZB_Urb_S = 1 .

IF ( SD_Pst = 3534 ) ZB_Urb_S = 1 .

IF ( SD_Pst = 3541 ) ZB_Urb_S = 1 .

IF ( SD_Pst = 3542 ) ZB_Urb_S = 1 .

IF ( SD_Pst = 3543 ) ZB_Urb_S = 1 .

IF ( SD_Pst = 3544 ) ZB_Urb_S = 1 .

IF ( SD_Pst = 3545 ) ZB_Urb_S = 1 .

IF ( SD_Pst = 3546 ) ZB_Urb_S = 1 .

IF ( SD_Pst = 3551 ) ZB_Urb_S = 1 .

IF ( SD_Pst = 3552 ) ZB_Urb_S = 1 .

IF ( SD_Pst = 3553 ) ZB_Urb_S = 1 .

IF ( SD_Pst = 3554 ) ZB_Urb_S = 1 .

IF ( SD_Pst = 3555 ) ZB_Urb_S = 1 .

IF ( SD_Pst = 3561 ) ZB_Urb_S = 1 .

IF ( SD_Pst = 3562 ) ZB_Urb_S = 1 .

IF ( SD_Pst = 3563 ) ZB_Urb_S = 1 .

IF ( SD_Pst = 3564 ) ZB_Urb_S = 1 .

IF ( SD_Pst = 3565 ) ZB_Urb_S = 1 .

IF ( SD_Pst = 3566 ) ZB_Urb_S = 1 .

IF ( SD_Pst = 3571 ) ZB_Urb_S = 1 .

IF ( SD_Pst = 3572 ) ZB_Urb_S = 1 .

IF ( SD_Pst = 3581 ) ZB_Urb_S = 1 .

IF ( SD_Pst = 3582 ) ZB_Urb_S = 1 .

IF ( SD_Pst = 3583 ) ZB_Urb_S = 1 .

IF ( SD_Pst = 3584 ) ZB_Urb_S = 1 .

IF ( SD_Pst = 3585 ) ZB_Urb_S = 1 .

IF ( SD_Pst = 3573 ) ZB_Urb_S = 1 .

IF ( SD_Pst = 1309 ) ZB_Urb_S = 2 .

IF ( SD_Pst = 1311 ) ZB_Urb_S = 2 .

IF ( SD_Pst = 1312 ) ZB_Urb_S = 2 .

IF ( SD_Pst = 1313 ) ZB_Urb_S = 2 .

IF ( SD_Pst = 1314 ) ZB_Urb_S = 2 .

IF ( SD_Pst = 1315 ) ZB_Urb_S = 2 .

IF ( SD_Pst = 1316 ) ZB_Urb_S = 2 .

IF ( SD_Pst = 1317 ) ZB_Urb_S = 2 .

IF ( SD_Pst = 1318 ) ZB_Urb_S = 2 .

IF ( SD_Pst = 1319 ) ZB_Urb_S = 2 .

IF ( SD_Pst = 1321 ) ZB_Urb_S = 2 .

IF ( SD_Pst = 1323 ) ZB_Urb_S = 2 .

IF ( SD_Pst = 1324 ) ZB_Urb_S = 2 .

IF ( SD_Pst = 1325 ) ZB_Urb_S = 2 .

IF ( SD_Pst = 1326 ) ZB_Urb_S = 2 .

IF ( SD_Pst = 1327 ) ZB_Urb_S = 2 .

IF ( SD_Pst = 1328 ) ZB_Urb_S = 2 .

IF ( SD_Pst = 1331 ) ZB_Urb_S = 2 .

IF ( SD_Pst = 1333 ) ZB_Urb_S = 2 .

IF ( SD_Pst = 1334 ) ZB_Urb_S = 2 .

IF ( SD_Pst = 1335 ) ZB_Urb_S = 2 .

IF ( SD_Pst = 1336 ) ZB_Urb_S = 2 .

IF ( SD_Pst = 1338 ) ZB_Urb_S = 2 .

IF ( SD_Pst = 1339 ) ZB_Urb_S = 2 .

IF ( SD_Pst = 1341 ) ZB_Urb_S = 2 .

IF ( SD_Pst = 1342 ) ZB_Urb_S = 2 .

IF ( SD_Pst = 1343 ) ZB_Urb_S = 2 .

IF ( SD_Pst = 1349 ) ZB_Urb_S = 2 .

IF ( SD_Pst = 1351 ) ZB_Urb_S = 2 .

IF ( SD_Pst = 1352 ) ZB_Urb_S = 2 .

IF ( SD_Pst = 1353 ) ZB_Urb_S = 2 .

IF ( SD_Pst = 1354 ) ZB_Urb_S = 2 .

IF ( SD_Pst = 1355 ) ZB_Urb_S = 2 .

IF ( SD_Pst = 1356 ) ZB_Urb_S = 2 .

IF ( SD_Pst = 1357 ) ZB_Urb_S = 2 .

IF ( SD_Pst = 1358 ) ZB_Urb_S = 2 .

IF ( SD_Pst = 1359 ) ZB_Urb_S = 2 .

IF ( SD_Pst = 1361 ) ZB_Urb_S = 2 .

IF ( SD_Pst = 1362 ) ZB_Urb_S = 2 .

IF ( SD_Pst = 4811 ) ZB_Urb_S = 2 .

IF ( SD_Pst = 4812 ) ZB_Urb_S = 2 .

IF ( SD_Pst = 4813 ) ZB_Urb_S = 2 .

IF ( SD_Pst = 4814 ) ZB_Urb_S = 2 .

IF ( SD_Pst = 4815 ) ZB_Urb_S = 2 .

IF ( SD_Pst = 4816 ) ZB_Urb_S = 2 .

IF ( SD_Pst = 4817 ) ZB_Urb_S = 2 .

IF ( SD_Pst = 4818 ) ZB_Urb_S = 2 .

IF ( SD_Pst = 4819 ) ZB_Urb_S = 2 .

IF ( SD_Pst = 4822 ) ZB_Urb_S = 2 .

IF ( SD_Pst = 4823 ) ZB_Urb_S = 2 .

IF ( SD_Pst = 4824 ) ZB_Urb_S = 2 .

IF ( SD_Pst = 4825 ) ZB_Urb_S = 2 .

IF ( SD_Pst = 4826 ) ZB_Urb_S = 2 .

IF ( SD_Pst = 4827 ) ZB_Urb_S = 2 .

IF ( SD_Pst = 4834 ) ZB_Urb_S = 2 .

IF ( SD_Pst = 4835 ) ZB_Urb_S = 2 .

IF ( SD_Pst = 4836 ) ZB_Urb_S = 2 .

IF ( SD_Pst = 4837 ) ZB_Urb_S = 2 .

IF ( SD_Pst = 4838 ) ZB_Urb_S = 2 .

IF ( SD_Pst = 4839 ) ZB_Urb_S = 2 .

IF ( SD_Pst = 4841 ) ZB_Urb_S = 2 .

IF ( SD_Pst = 4847 ) ZB_Urb_S = 2 .

IF ( SD_Pst = 4851 ) ZB_Urb_S = 2 .

IF ( SD_Pst = 4854 ) ZB_Urb_S = 2 .

IF ( SD_Pst = 5011 ) ZB_Urb_S = 2 .

IF ( SD_Pst = 5012 ) ZB_Urb_S = 2 .

IF ( SD_Pst = 5013 ) ZB_Urb_S = 2 .

IF ( SD_Pst = 5014 ) ZB_Urb_S = 2 .

IF ( SD_Pst = 5015 ) ZB_Urb_S = 2 .

IF ( SD_Pst = 5017 ) ZB_Urb_S = 2 .

IF ( SD_Pst = 5018 ) ZB_Urb_S = 2 .

IF ( SD_Pst = 5021 ) ZB_Urb_S = 2 .

IF ( SD_Pst = 5022 ) ZB_Urb_S = 2 .

IF ( SD_Pst = 5025 ) ZB_Urb_S = 2 .

IF ( SD_Pst = 5026 ) ZB_Urb_S = 2 .

IF ( SD_Pst = 5032 ) ZB_Urb_S = 2 .

IF ( SD_Pst = 5035 ) ZB_Urb_S = 2 .

IF ( SD_Pst = 5036 ) ZB_Urb_S = 2 .

IF ( SD_Pst = 5037 ) ZB_Urb_S = 2 .

IF ( SD_Pst = 5038 ) ZB_Urb_S = 2 .

IF ( SD_Pst = 5041 ) ZB_Urb_S = 2 .

IF ( SD_Pst = 5042 ) ZB_Urb_S = 2 .

IF ( SD_Pst = 5043 ) ZB_Urb_S = 2 .

IF ( SD_Pst = 5044 ) ZB_Urb_S = 2 .

IF ( SD_Pst = 5046 ) ZB_Urb_S = 2 .

IF ( SD_Pst = 5047 ) ZB_Urb_S = 2 .

IF ( SD_Pst = 5045 ) ZB_Urb_S = 2 .

IF ( SD_Pst = 5048 ) ZB_Urb_S = 2 .

IF ( SD_Pst = 5049 ) ZB_Urb_S = 2 .

IF ( SD_Pst = 5056 ) ZB_Urb_S = 2 .

IF ( SD_Pst = 5071 ) ZB_Urb_S = 2 .

IF ( SD_Pst = 5611 ) ZB_Urb_S = 2 .

IF ( SD_Pst = 5612 ) ZB_Urb_S = 2 .

IF ( SD_Pst = 5613 ) ZB_Urb_S = 2 .

IF ( SD_Pst = 5614 ) ZB_Urb_S = 2 .

IF ( SD_Pst = 5615 ) ZB_Urb_S = 2 .

IF ( SD_Pst = 5616 ) ZB_Urb_S = 2 .

IF ( SD_Pst = 5621 ) ZB_Urb_S = 2 .

IF ( SD_Pst = 5622 ) ZB_Urb_S = 2 .

IF ( SD_Pst = 5623 ) ZB_Urb_S = 2 .

IF ( SD_Pst = 5624 ) ZB_Urb_S = 2 .

IF ( SD_Pst = 5625 ) ZB_Urb_S = 2 .

IF ( SD_Pst = 5626 ) ZB_Urb_S = 2 .

IF ( SD_Pst = 5627 ) ZB_Urb_S = 2 .

IF ( SD_Pst = 5628 ) ZB_Urb_S = 2 .

IF ( SD_Pst = 5629 ) ZB_Urb_S = 2 .

IF ( SD_Pst = 5631 ) ZB_Urb_S = 2 .

IF ( SD_Pst = 5632 ) ZB_Urb_S = 2 .

IF ( SD_Pst = 5633 ) ZB_Urb_S = 2 .

IF ( SD_Pst = 5641 ) ZB_Urb_S = 2 .

IF ( SD_Pst = 5642 ) ZB_Urb_S = 2 .

IF ( SD_Pst = 5643 ) ZB_Urb_S = 2 .

IF ( SD_Pst = 5644 ) ZB_Urb_S = 2 .

IF ( SD_Pst = 5645 ) ZB_Urb_S = 2 .

IF ( SD_Pst = 5646 ) ZB_Urb_S = 2 .

IF ( SD_Pst = 5647 ) ZB_Urb_S = 2 .

IF ( SD_Pst = 5651 ) ZB_Urb_S = 2 .

IF ( SD_Pst = 5652 ) ZB_Urb_S = 2 .

IF ( SD_Pst = 5653 ) ZB_Urb_S = 2 .

IF ( SD_Pst = 5654 ) ZB_Urb_S = 2 .

IF ( SD_Pst = 5655 ) ZB_Urb_S = 2 .

IF ( SD_Pst = 5656 ) ZB_Urb_S = 2 .

IF ( SD_Pst = 5657 ) ZB_Urb_S = 2 .

IF ( SD_Pst = 5658 ) ZB_Urb_S = 2 .

IF ( SD_Pst = 6511 ) ZB_Urb_S = 2 .

IF ( SD_Pst = 6512 ) ZB_Urb_S = 2 .

IF ( SD_Pst = 6521 ) ZB_Urb_S = 2 .

IF ( SD_Pst = 6522 ) ZB_Urb_S = 2 .

IF ( SD_Pst = 6523 ) ZB_Urb_S = 2 .

IF ( SD_Pst = 6524 ) ZB_Urb_S = 2 .

IF ( SD_Pst = 6525 ) ZB_Urb_S = 2 .

IF ( SD_Pst = 6531 ) ZB_Urb_S = 2 .

IF ( SD_Pst = 6532 ) ZB_Urb_S = 2 .

IF ( SD_Pst = 6533 ) ZB_Urb_S = 2 .

IF ( SD_Pst = 6534 ) ZB_Urb_S = 2 .

IF ( SD_Pst = 6535 ) ZB_Urb_S = 2 .

IF ( SD_Pst = 6536 ) ZB_Urb_S = 2 .

IF ( SD_Pst = 6537 ) ZB_Urb_S = 2 .

IF ( SD_Pst = 6538 ) ZB_Urb_S = 2 .

IF ( SD_Pst = 6541 ) ZB_Urb_S = 2 .

IF ( SD_Pst = 6542 ) ZB_Urb_S = 2 .

IF ( SD_Pst = 6543 ) ZB_Urb_S = 2 .

IF ( SD_Pst = 6544 ) ZB_Urb_S = 2 .

IF ( SD_Pst = 6545 ) ZB_Urb_S = 2 .

IF ( SD_Pst = 6546 ) ZB_Urb_S = 2 .

IF ( SD_Pst = 6663 ) ZB_Urb_S = 2 .

IF ( SD_Pst = 9711 ) ZB_Urb_S = 2 .

IF ( SD_Pst = 9712 ) ZB_Urb_S = 2 .

IF ( SD_Pst = 9713 ) ZB_Urb_S = 2 .

IF ( SD_Pst = 9714 ) ZB_Urb_S = 2 .

IF ( SD_Pst = 9715 ) ZB_Urb_S = 2 .

IF ( SD_Pst = 9716 ) ZB_Urb_S = 2 .

IF ( SD_Pst = 9717 ) ZB_Urb_S = 2 .

IF ( SD_Pst = 9718 ) ZB_Urb_S = 2 .

IF ( SD_Pst = 9721 ) ZB_Urb_S = 2 .

IF ( SD_Pst = 9722 ) ZB_Urb_S = 2 .

IF ( SD_Pst = 9723 ) ZB_Urb_S = 2 .

IF ( SD_Pst = 9724 ) ZB_Urb_S = 2 .

IF ( SD_Pst = 9725 ) ZB_Urb_S = 2 .

IF ( SD_Pst = 9726 ) ZB_Urb_S = 2 .

IF ( SD_Pst = 9727 ) ZB_Urb_S = 2 .

IF ( SD_Pst = 9728 ) ZB_Urb_S = 2 .

IF ( SD_Pst = 9731 ) ZB_Urb_S = 2 .

IF ( SD_Pst = 9732 ) ZB_Urb_S = 2 .

IF ( SD_Pst = 9733 ) ZB_Urb_S = 2 .

IF ( SD_Pst = 9734 ) ZB_Urb_S = 2 .

IF ( SD_Pst = 9735 ) ZB_Urb_S = 2 .

IF ( SD_Pst = 9736 ) ZB_Urb_S = 2 .

IF ( SD_Pst = 9737 ) ZB_Urb_S = 2 .

IF ( SD_Pst = 9738 ) ZB_Urb_S = 2 .

IF ( SD_Pst = 9741 ) ZB_Urb_S = 2 .

IF ( SD_Pst = 9742 ) ZB_Urb_S = 2 .

IF ( SD_Pst = 9743 ) ZB_Urb_S = 2 .

IF ( SD_Pst = 9744 ) ZB_Urb_S = 2 .

IF ( SD_Pst = 9745 ) ZB_Urb_S = 2 .

IF ( SD_Pst = 9746 ) ZB_Urb_S = 2 .

IF SYSMIS( SD_Pst) ZB_Urb_S = 999 .

VARIABLE LABELS ZB_Urb_S 'verdeling aantal ziekenhuizen per postcode gebied, cat 3 SYSMIS'.

VALUE LABELS ZB_Urb_S

1 '4 grote steden'

2 '5-10 grootste steden'

3 '>10'

999 'missing'.

FREQUENCIES ZB_Urb_S.

NUMERIC ZB_Urb_M (F2.0).

COMPUTE ZB_Urb_M = ZB_Urb_S.

VARIABLE LABELS ZB_Urb_M 'verdeling aantal ziekenhuizen per postcode gebied, cat 3 Missing'.

VALUE LABELS ZB_Urb_M

1 '4 grote steden'

2 '5-10 grootste steden'

3 '>10'

999 'missing'.

MISSING VALUES ZB_Urb_M (999).

FREQUENCIES ZB_Urb_M.

*REISTIJD.

* Reistijd is eerder in seconden gecodeerd, naar de 5 dichtsbijzijnde ziekenhuizen, daarboven alles waarde 5000sec gegeven.

* Nu dichtomiseren in groter en kleiner dan 20 minuten. Zie publicaties AMC.

NUMERIC ZB_Ret_S_Cat2 (F2.0).

COMPUTE ZB_Ret_S_Cat2 =-999.

IF (ZB_Peb_S=1) ZB_Ret_S_Cat2=1.

IF (ZB_Peb_S=2 OR ZB_Peb_S=3) ZB_Ret_S_Cat2=2.

IF (ZB_Peb_S=999) ZB_Ret_S_Cat2 =999.

VARIABLE LABELS ZB_Ret_S_Cat2 'Reistijd naar ziekenhuis, Cat2 SYSMIS'.

VALUE LABELS ZB_Ret_S_Cat2

1 'Geen reistijd'

2 'Reistijd'

999 'Missing'.

FREQUENCIES ZB_Ret_S_Cat2.

NUMERIC ZB_Ret_M_Cat2 (F2.0).

COMPUTE ZB_Ret_M_Cat2 =-999.

IF (ZB_Peb_S=1) ZB_Ret_M_Cat2=1.

IF (ZB_Peb_S=2 OR ZB_Peb_S=3) ZB_Ret_M_Cat2=2.

IF (ZB_Peb_S=999) ZB_Ret_M_Cat2 =999.

VARIABLE LABELS ZB_Ret_M_Cat2 'Reistijd naar ziekenhuis, Cat2 SYSMIS'.

VALUE LABELS ZB_Ret_M_Cat2

1 'Geen reistijd'

2 'Reistijd'

999 'Missing'.

MISSING VALUES ZB_Ret_M_Cat2 (999).

FREQUENCIES ZB_Ret_M_Cat2.

NUMERIC ZB_Art_S_Cat3 (F2.0).

COMPUTE ZB_Art_S_Cat3 =-999.

IF (ZB_Art_S<=1200) ZB_Art_S_Cat3 =2.

IF (ZB_Art_S>1200) ZB_Art_S_Cat3 =3.

IF (ZB_Peb_S=1) ZB_Art_S_Cat3=1.

IF (SYSMIS(ZB_Art_S)) ZB_Art_S_Cat3 =999.

VARIABLE LABELS ZB_Art_S_Cat3 'Reistijd naar ziekenhuis, Cat2 SYSMIS'.

VALUE LABELS ZB_Art_S_Cat3

1 'Geen reistijd'

2 'Reistijd ≤20 minuten (1200 seconden) '

3 'Reistijd >20 minuten (1200 seconden) '

999 'Missing'.

FREQUENCIES ZB_Art_S_Cat3.

NUMERIC ZB_Art_S_Cat6 (F2.0).

COMPUTE ZB_Art_S_Cat6 =-999.

IF (ZB_Art_S<=1200 AND PB_Emb_S_Cat2=1) ZB_Art_S_Cat6 =3.

IF (ZB_Art_S<=1200 AND PB_Emb_S_Cat2=2) ZB_Art_S_Cat6 =4.

IF (ZB_Art_S>1200 AND PB_Emb_S_Cat2=1) ZB_Art_S_Cat6 =5.

IF (ZB_Art_S>1200 AND PB_Emb_S_Cat2=2) ZB_Art_S_Cat6 =6.

IF (ZB_Peb_S=1 AND PB_Emb_S_Cat2=1) ZB_Art_S_Cat6=1.

IF (ZB_Peb_S=1 AND PB_Emb_S_Cat2=2) ZB_Art_S_Cat6=2.

IF (SYSMIS(ZB_Art_S)) ZB_Art_S_Cat6 =999.

VARIABLE LABELS ZB_Art_S_Cat6 'Reistijd naar ziekenhuis, Cat6 SYSMIS'.

VALUE LABELS ZB_Art_S_Cat6

1 'Geen reistijd, moeder en kind gezond'

2 'Geen reistijd, moeder en/of kind ongezond'

3 'Reistijd ≤20 minuten (1200 seconden), moeder en kind gezond'

4 'Reistijd ≤20 minuten (1200 seconden), moeder en/of kind ongezond'

5 'Reistijd >20 minuten (1200 seconden), moeder en kind gezond '

6 'Reistijd >20 minuten (1200 seconden), moeder en/of kind ongezond'

999 'Missing'.

FREQUENCIES ZB_Art_S_Cat6.

NUMERIC ZB_Art_M_Cat2 (F2.0).

COMPUTE ZB_Art_M_Cat2 =-999.

IF (ZB_Art_S<=1200) ZB_Art_M_Cat2 =1.

IF (ZB_Art_S>1200) ZB_Art_M_Cat2 =2.

IF (SYSMIS(ZB_Art_S)) ZB_Art_M_Cat2 =999.

VARIABLE LABELS ZB_Art_M_Cat2 'Reistijd naar ziekenhuis, Cat2 Missing'.

VALUE LABELS ZB_Art_M_Cat2

1 'Reistijd ≤20 minuten (1200 seconden) '

2 'Reistijd >20 minuten (1200 seconden) '

999 'Missing'.

MISSING VALUES ZB_Art_M_Cat2 (999).

FREQUENCIES ZB_Art_M_Cat2.

*AANTAL PARTUSSEN PER JAAR.

NUMERIC VSV_GR_S (F2.0).

COMPUTE VSV_GR_S = 2.

IF (VSV=5 OR VSV=10 OR VSV=17 OR VSV=24 OR VSV=26 OR VSV=35 OR VSV=42

OR VSV=51 OR VSV=56 OR VSV=60 OR VSV=64 OR VSV=73 OR VSV=80 OR VSV=81

OR VSV=86 OR VSV=90 OR VSV=91 OR VSV=92) VSV_GR_S=1.

IF (VSV=1 OR VSV=3 OR VSV=4 OR VSV=9 OR VSV=13 OR VSV=15 OR VSV=20

OR VSV=22 OR VSV=29 OR VSV=31 OR VSV=34 OR VSV=39 OR VSV=40 OR VSV=46

OR VSV=50 OR VSV=55 OR VSV=67 OR VSV=71 OR VSV=75 OR VSV=82 OR VSV=85 OR VSV=87 OR VSV=97) VSV_GR_S=3.

IF (SYSMIS(VSV) OR VSV=98 OR VSV=100) VSV_GR_S = 999.

VARIABLE LABELS VSV_GR_S 'grootte van het ziekenhuis/vsv'.

VALUE LABELS VSV_GR_S

1 '1e percentiel (<750 partussen per jaar)'

2 '2e en 3e percentiel'

3 '4e percentiel (>1500 partussen per jaar)'

999 'Missing'.

FREQUENCIES VSV_GR_S.

NUMERIC VSV_GR_M (F2.0).

COMPUTE VSV_GR_M = 2.

IF (VSV=5 OR VSV=10 OR VSV=17 OR VSV=24 OR VSV=26 OR VSV=35 OR VSV=42

OR VSV=51 OR VSV=56 OR VSV=60 OR VSV=64 OR VSV=73 OR VSV=80 OR VSV=81

OR VSV=86 OR VSV=90 OR VSV=91 OR VSV=92) VSV_GR_M=1.

IF (VSV=1 OR VSV=3 OR VSV=4 OR VSV=9 OR VSV=13 OR VSV=15 OR VSV=20

OR VSV=22 OR VSV=29 OR VSV=31 OR VSV=34 OR VSV=39 OR VSV=40 OR VSV=46

OR VSV=50 OR VSV=55 OR VSV=67 OR VSV=71 OR VSV=75 OR VSV=82 OR VSV=85 OR VSV=87 OR VSV=97) VSV_GR_M=3.

IF (SYSMIS(VSV) OR VSV=98 OR VSV=100) VSV_GR_M = 999.

VARIABLE LABELS VSV_GR_M 'grootte van het ziekenhuis/vsv, Missing'.

VALUE LABELS VSV_GR_M

1 '1e percentiel (<750 partussen per jaar)'

2 '2e en 3e percentiel'

3 '4e percentiel (>1500 partussen per jaar)'

999 'Missing'.

MISSING VALUES VSV_GR_M (999).

FREQUENCIES VSV_GR_M.

# Test-retest reliability MEAN

*RESPECT.

*ICC.

RELIABILITY

/VARIABLES=RQ_R_Dom_A RQ_R_Re_Dom_A

/SCALE('ICC Respect') ALL

/MODEL=ALPHA

/STATISTICS=DESCRIPTIVE SCALE

/ICC=MODEL(MIXED) TYPE(ABSOLUTE) CIN=95 TESTVAL=0.

*AUTONOMIE.

RELIABILITY

/VARIABLES=RQ_A_Dom_A RQ_A_Re_Dom_A

/SCALE('ICC Autonomie') ALL

/MODEL=ALPHA

/STATISTICS=DESCRIPTIVE SCALE

/ICC=MODEL(MIXED) TYPE(ABSOLUTE) CIN=95 TESTVAL=0.

*PRIVACY.

RELIABILITY

/VARIABLES=RQ_P_Dom_A RQ_P_Re_Dom_A

/SCALE('ICC Privacy') ALL

/MODEL=ALPHA

/STATISTICS=DESCRIPTIVE SCALE

/ICC=MODEL(MIXED) TYPE(ABSOLUTE) CIN=95 TESTVAL=0.

*COMMUNCIATION.

RELIABILITY

/VARIABLES=RQ_C_Dom_A RQ_C_Re_Dom_A

/SCALE('ICC Communicatie') ALL

/MODEL=ALPHA

/STATISTICS=DESCRIPTIVE SCALE

/ICC=MODEL(MIXED) TYPE(ABSOLUTE) CIN=95 TESTVAL=0.

*TIJD TOT GEBODEN HULP.

RELIABILITY

/VARIABLES=RQ_T_Dom_A RQ_T_Re_Dom_A

/SCALE('ICC Tyd tot hulp') ALL

/MODEL=ALPHA

/STATISTICS=DESCRIPTIVE SCALE

/ICC=MODEL(MIXED) TYPE(ABSOLUTE) CIN=95 TESTVAL=0.

*SOCIALE ONDERSTEUNING.

RELIABILITY

/VARIABLES=RQ_S_Dom_A RQ_S_Re_Dom_A

/SCALE('ICC Soc. Ondersteuning') ALL

/MODEL=ALPHA

/STATISTICS=DESCRIPTIVE SCALE

/ICC=MODEL(MIXED) TYPE(ABSOLUTE) CIN=95 TESTVAL=0.

*FACILITEITEN.

RELIABILITY

/VARIABLES=RQ_F_Dom_A RQ_F_Re_Dom_A

/SCALE('ICC Faciliteiten') ALL

/MODEL=ALPHA

/STATISTICS=DESCRIPTIVE SCALE

/ICC=MODEL(MIXED) TYPE(ABSOLUTE) CIN=95 TESTVAL=0.

*KEUZE EN CONTINUITEIT.

RELIABILITY

/VARIABLES=RQ_K_Dom_A RQ_K_Re_Dom_A

/SCALE('ICC Keuze en Continuiteit') ALL

/MODEL=ALPHA

/STATISTICS=DESCRIPTIVE SCALE

/ICC=MODEL(MIXED) TYPE(ABSOLUTE) CIN=95 TESTVAL=0.

*PERSOONSGEBONDEN DOMEINEN.

RELIABILITY

/VARIABLES=RQ_PS_Dom_A RQ_PS_Dom_A_Re

/SCALE('ICC persoons domeinen') ALL

/MODEL=ALPHA

/STATISTICS=DESCRIPTIVE SCALE

/ICC=MODEL(MIXED) TYPE(ABSOLUTE) CIN=95 TESTVAL=0.

*SETTINGGEBONDEN DOMEINEN.

RELIABILITY

/VARIABLES=RQ_ST_Dom_A RQ_ST_Dom_A_Re

/SCALE('ICC setting domeinen') ALL

/MODEL=ALPHA

/STATISTICS=DESCRIPTIVE SCALE

/ICC=MODEL(MIXED) TYPE(ABSOLUTE) CIN=95 TESTVAL=0.

*OVERAL SCORE.

RELIABILITY

/VARIABLES=RQ_EvT_A RQ_EvT_Re_A

/SCALE('ICC Overall score') ALL

/MODEL=ALPHA

/STATISTICS=DESCRIPTIVE SCALE

/ICC=MODEL(MIXED) TYPE(ABSOLUTE) CIN=95 TESTVAL=0.

* STAP 2: blant altman plot:

*Bias: test-hertest --> gem.

NUMERIC RQ_R_Vth (F2.0).

COMPUTE RQ_R_Vth = RQ_R_Dom_A - RQ_R_Re_Dom_A.

VARIABLE LABELS RQ_R_Vth 'Difference test-rertest - Dignity'.

DESCRIPTIVES RQ_R_Vth

/STATISTICS MEAN STDDEV.

NUMERIC RQ_A_Vth (F2.0).

COMPUTE RQ_A_Vth = RQ_A_Dom_A - RQ_A_Re_Dom_A.

VARIABLE LABELS RQ_A_Vth 'Difference test-rertest - Autonomy'.

DESCRIPTIVES RQ_A_Vth

/STATISTICS MEAN STDDEV.

NUMERIC RQ_P_Vth (F2.0).

COMPUTE RQ_P_Vth = RQ_P_Dom_A - RQ_P_Re_Dom_A.

VARIABLE LABELS RQ_P_Vth 'Difference test-rertest - Confidentiality'.

DESCRIPTIVES RQ_P_Vth

/STATISTICS MEAN STDDEV.

NUMERIC RQ_C_Vth (F2.0).

COMPUTE RQ_C_Vth = RQ_C_Dom_A - RQ_C_Re_Dom_A.

VARIABLE LABELS RQ_C_Vth 'Difference test-rertest - Communication'.

DESCRIPTIVES RQ_C_Vth

/STATISTICS MEAN STDDEV.

NUMERIC RQ_T_Vth (F2.0).

COMPUTE RQ_T_Vth = RQ_T_Dom_A - RQ_T_Re_Dom_A.

VARIABLE LABELS RQ_T_Vth 'Difference test-rertest - Prompt Attention'.

DESCRIPTIVES RQ_T_Vth

/STATISTICS MEAN STDDEV.

NUMERIC RQ_S_Vth (F2.0).

COMPUTE RQ_S_Vth = RQ_S_Dom_A - RQ_S_Re_Dom_A.

VARIABLE LABELS RQ_S_Vth 'Difference test-rertest - Social Consideration'.

DESCRIPTIVES RQ_S_Vth

/STATISTICS MEAN STDDEV.

NUMERIC RQ_F_Vth (F2.0).

COMPUTE RQ_F_Vth = RQ_F_Dom_A - RQ_F_Re_Dom_A.

VARIABLE LABELS RQ_F_Vth 'Difference test-rertest - Basic Amenities'.

DESCRIPTIVES RQ_R_Vth

/STATISTICS MEAN STDDEV.

NUMERIC RQ_K_Vth (F2.0).

COMPUTE RQ_K_Vth = RQ_K_Dom_A - RQ_K_Re_Dom_A.

VARIABLE LABELS RQ_K_Vth 'Difference test-rertest - Choice and continuity'.

DESCRIPTIVES RQ_K_Vth

/STATISTICS MEAN STDDEV.

NUMERIC RQ_PS_Vth (F2.2).

COMPUTE RQ_PS_Vth = RQ_PS_Dom_A - RQ_PS_Dom_A_Re.

VARIABLE LABELS RQ_PS_Vth 'Difference test-rertest - personal score'.

DESCRIPTIVES RQ_PS_Vth

/STATISTICS MEAN STDDEV.

NUMERIC RQ_ST_Vth (F2.2).

COMPUTE RQ_ST_Vth = RQ_ST_Dom_A - RQ_ST_Dom_A_Re.

VARIABLE LABELS RQ_ST_Vth 'Difference test-rertest - Setting score'.

DESCRIPTIVES RQ_ST_Vth

/STATISTICS MEAN STDDEV.

NUMERIC RQ_EvT_Vth (F2.2).

COMPUTE RQ_EvT_Vth = RQ_EvT_A - RQ_EvT_Re_A.

VARIABLE LABELS RQ_EvT_Vth 'Difference test-rertest - Overall score'.

DESCRIPTIVES RQ_EvT_Vth

/STATISTICS MEAN STDDEV.

* 1/2(test+hertest).

NUMERIC RQ_R_BAP_Re_X (F2.0).

COMPUTE RQ_R_BAP_Re_X =(0.5*(RQ_R_Dom_A + RQ_R_Re_Dom_A)).

IF (MISSING(RQ_R_Dom_A) OR MISSING(RQ_R_Re_Dom_A)) RQ_R_BAP_Re_X=$SYSMIS.

VARIABLE LABELS RQ_R_BAP_Re_X '1/2*Sum test-retest - Dignity'.

FREQUENCIES RQ_R_BAP_Re_X

/STATISTICS MEAN STDDEV.

NUMERIC RQ_A_BAP_Re_X (F2.0).

COMPUTE RQ_A_BAP_Re_X =(0.5*(RQ_A_Dom_A + RQ_A_Re_Dom_A)).

IF (MISSING(RQ_A_Dom_A) OR MISSING(RQ_A_Re_Dom_A)) RQ_A_BAP_Re_X=$SYSMIS.

VARIABLE LABELS RQ_A_BAP_Re_X '1/2*Sum test-retest Autonomy'.

FREQUENCIES RQ_A_BAP_Re_X

/STATISTICS MEAN STDDEV.

NUMERIC RQ_P_BAP_Re_X (F2.0).

COMPUTE RQ_P_BAP_Re_X =(0.5*(RQ_P_Dom_A + RQ_P_Re_Dom_A)).

IF (MISSING(RQ_P_Dom_A) OR MISSING(RQ_P_Re_Dom_A)) RQ_P_BAP_Re_X=$SYSMIS.

VARIABLE LABELS RQ_P_BAP_Re_X '1/2*Sum test-retest Condidentiality'.

FREQUENCIES RQ_P_BAP_Re_X

/STATISTICS MEAN STDDEV.

NUMERIC RQ_C_BAP_Re_X (F2.0).

COMPUTE RQ_C_BAP_Re_X =(0.5*(RQ_C_Dom_A + RQ_C_Re_Dom_A)).

IF (MISSING(RQ_C_Dom_A) OR MISSING(RQ_C_Re_Dom_A)) RQ_C_BAP_Re_X=$SYSMIS.

VARIABLE LABELS RQ_C_BAP_Re_X '1/2*Sum test-retest Communication'.

FREQUENCIES RQ_C_BAP_Re_X

/STATISTICS MEAN STDDEV.

NUMERIC RQ_T_BAP_Re_X (F2.0).

COMPUTE RQ_T_BAP_Re_X =(0.5*(RQ_T_Dom_A + RQ_T_Re_Dom_A)).

IF (MISSING(RQ_T_Dom_A) OR MISSING(RQ_T_Re_Dom_A)) RQ_T_BAP_Re_X=$SYSMIS.

VARIABLE LABELS RQ_T_BAP_Re_X '1/2*Sum test-retest Prompt Attention'.

FREQUENCIES RQ_T_BAP_Re_X

/STATISTICS MEAN STDDEV.

NUMERIC RQ_S_BAP_Re_X (F2.0).

COMPUTE RQ_S_BAP_Re_X =(0.5*(RQ_S_Dom_A + RQ_S_Re_Dom_A)).

IF (MISSING(RQ_S_Dom_A) OR MISSING(RQ_S_Re_Dom_A)) RQ_S_BAP_Re_X=$SYSMIS.

VARIABLE LABELS RQ_S_BAP_Re_X '1/2*Sum test-retest Social Consideration'.

FREQUENCIES RQ_S_BAP_Re_X

/STATISTICS MEAN STDDEV.

NUMERIC RQ_F_BAP_Re_X (F2.0).

COMPUTE RQ_F_BAP_Re_X =(0.5*(RQ_F_Dom_A + RQ_F_Re_Dom_A)).

IF (MISSING(RQ_F_Dom_A) OR MISSING(RQ_F_Re_Dom_A)) RQ_F_BAP_Re_X=$SYSMIS.

VARIABLE LABELS RQ_F_BAP_Re_X '1/2*Sum test-retest Basic Amenities'.

FREQUENCIES RQ_F_BAP_Re_X

/STATISTICS MEAN STDDEV.

NUMERIC RQ_K_BAP_Re_X (F2.0).

COMPUTE RQ_K_BAP_Re_X =(0.5*(RQ_K_Dom_A + RQ_K_Re_Dom_A)).

IF (MISSING(RQ_K_Dom_A) OR MISSING(RQ_K_Re_Dom_A)) RQ_K_BAP_Re_X=$SYSMIS.

VARIABLE LABELS RQ_K_BAP_Re_X '1/2*Sum test-retest Choice and Continuity'.

FREQUENCIES RQ_K_BAP_Re_X

/STATISTICS MEAN STDDEV.

NUMERIC RQ_EvT_BAP_Re_X (F2.0).

COMPUTE RQ_EvT_BAP_Re_X =(0.5*(RQ_EvT_A + RQ_EvT_Re_A)).

IF (MISSING(RQ_EvT_A) OR MISSING(RQ_EvT_Re_A)) RQ_EvT_BAP_Re_X=$SYSMIS.

VARIABLE LABELS RQ_EvT_BAP_Re_X '1/2*Sum test-retest Overall score'.

FREQUENCIES RQ_EvT_BAP_Re_X

/STATISTICS MEAN STDDEV.

*Scatterplot.

GRAPH

/SCATTERPLOT(BIVAR)=RQ_R_BAP_Re_X WITH RQ_R_Vth

/MISSING=LISTWISE.

GRAPH

/SCATTERPLOT(BIVAR)=RQ_A_BAP_Re_X WITH RQ_A_Vth

/MISSING=LISTWISE.

GRAPH

/SCATTERPLOT(BIVAR)=RQ_P_BAP_Re_X WITH RQ_P_Vth

/MISSING=LISTWISE.

GRAPH

/SCATTERPLOT(BIVAR)=RQ_C_BAP_Re_X WITH RQ_C_Vth

/MISSING=LISTWISE.

GRAPH

/SCATTERPLOT(BIVAR)=RQ_T_BAP_Re_X WITH RQ_T_Vth

/MISSING=LISTWISE.

GRAPH

/SCATTERPLOT(BIVAR)=RQ_S_BAP_Re_X WITH RQ_S_Vth

/MISSING=LISTWISE.

GRAPH

/SCATTERPLOT(BIVAR)=RQ_F_BAP_Re_X WITH RQ_F_Vth

/MISSING=LISTWISE.

GRAPH

/SCATTERPLOT(BIVAR)=RQ_K_BAP_Re_X WITH RQ_K_Vth

/MISSING=LISTWISE.

GRAPH

/SCATTERPLOT(BIVAR)=RQ_EvT_BAP_Re_X WITH RQ_EvT_Vth

/MISSING=LISTWISE.

* STAP 4: % Negatief, kappa.

*ALTERNATIEF 1: 1X NOOIT.

*Respect.

SORT CASES BY F_TH_Res.

FILTER BY F_TH_Res.

FREQUENCIES RQ_R_Neg_A_A1 RQ_R_Neg_A_Re_A1.

FILTER OFF.

CROSSTABS TABLES = RQ_R_Neg_A_A1 BY RQ_R_Neg_A_Re_A1

/CELLS COUNT ROW EXPECTED COLUMN RESID

/STATISTICS KAPPA.

*Autonomie.

SORT CASES BY F_TH_Res.

FILTER BY F_TH_Res.

FREQUENCIES RQ_A_Neg_A_A1 RQ_A_Neg_A_Re_A1.

FILTER OFF.

CROSSTABS TABLES = RQ_A_Neg_A_A1 BY RQ_A_Neg_A_Re_A1

/CELLS COUNT ROW EXPECTED COLUMN RESID

/STATISTICS KAPPA.

*Privacy.

SORT CASES BY F_TH_Res.

FILTER BY F_TH_Res.

FREQUENCIES RQ_P_Neg_A_A1 RQ_P_Neg_A_Re_A1.

FILTER OFF.

CROSSTABS TABLES = RQ_P_Neg_A_A1 BY RQ_P_Neg_A_Re_A1

/CELLS COUNT ROW EXPECTED COLUMN RESID

/STATISTICS KAPPA.

*Communicatie.

SORT CASES BY F_TH_Res.

FILTER BY F_TH_Res.

FREQUENCIES RQ_C_Neg_A_A1 RQ_C_Neg_A_Re_A1.

FILTER OFF.

CROSSTABS TABLES = RQ_C_Neg_A_A1 BY RQ_C_Neg_A_Re_A1

/CELLS COUNT ROW EXPECTED COLUMN RESID

/STATISTICS KAPPA.

*Tijd tot geboden hulp.

SORT CASES BY F_TH_Res.

FILTER BY F_TH_Res.

FREQUENCIES RQ_T_Neg_A_A1 RQ_T_Neg_A_Re_A1.

FILTER OFF.

CROSSTABS TABLES = RQ_T_Neg_A_A1 BY RQ_T_Neg_A_Re_A1

/CELLS COUNT ROW EXPECTED COLUMN RESID

/STATISTICS KAPPA.

*Sociale ondersteuning.

SORT CASES BY F_TH_Res.

FILTER BY F_TH_Res.

FREQUENCIES RQ_S_Neg_A_A1 RQ_S_Neg_A_Re_A1.

FILTER OFF.

CROSSTABS TABLES = RQ_S_Neg_A_A1 BY RQ_S_Neg_A_Re_A1

/CELLS COUNT ROW EXPECTED COLUMN RESID

/STATISTICS KAPPA.

*Faciliteiten.

SORT CASES BY F_TH_Res.

FILTER BY F_TH_Res.

FREQUENCIES RQ_F_Neg_A_A1 RQ_F_Neg_A_Re_A1.

FILTER OFF.

CROSSTABS TABLES = RQ_F_Neg_A_A1 BY RQ_F_Neg_A_Re_A1

/CELLS COUNT ROW EXPECTED COLUMN RESID

/STATISTICS KAPPA.

*Keuze en continuiteit.

SORT CASES BY F_TH_Res.

FILTER BY F_TH_Res.

FREQUENCIES RQ_K_Neg_A_A1 RQ_K_Neg_A_Re_A1.

FILTER OFF.

CROSSTABS TABLES = RQ_K_Neg_A_A1 BY RQ_K_Neg_A_Re_A1

/CELLS COUNT ROW EXPECTED COLUMN RESID

/STATISTICS KAPPA.

*Personal.

SORT CASES BY F_TH_Res.

FILTER BY F_TH_Res.

FREQUENCIES RQ_PS_Neg_A_A1 RQ_PS_Neg_A_RE_A1.

FILTER OFF.

CROSSTABS TABLES = RQ_PS_Neg_A_A1 BY RQ_PS_Neg_A_RE_A1

/CELLS COUNT ROW EXPECTED COLUMN RESID

/STATISTICS KAPPA.

*Setting.

SORT CASES BY F_TH_Res.

FILTER BY F_TH_Res.

FREQUENCIES RQ_ST_Neg_A_A1 RQ_ST_Neg_A_RE_A1.

FILTER OFF.

CROSSTABS TABLES = RQ_ST_Neg_A_A1 BY RQ_ST_Neg_A_RE_A1

/CELLS COUNT ROW EXPECTED COLUMN RESID

/STATISTICS KAPPA.

*overall score.

SORT CASES BY F_TH_Res.

FILTER BY F_TH_Res.

FREQUENCIES RQ_EvT_Neg_A_A1 RQ_EvT_Neg_A_RE_A1.

FILTER OFF.

CROSSTABS TABLES = RQ_EvT_Neg_A_A1 BY RQ_EvT_Neg_A_RE_A1

/CELLS COUNT ROW EXPECTED COLUMN RESID

/STATISTICS KAPPA.

* STAP 5: % overeenstemming in % negatief .

NUMERIC RQ_R_Oth_N_Cat2 (F2.0).

COMPUTE RQ_R_Oth_N_Cat2 = -999.

IF (RQ_R_Neg_A_A1 = RQ_R_Neg_A_Re_A1) RQ_R_Oth_N_Cat2 =1.

IF (RQ_R_Neg_A_A1 <> RQ_R_Neg_A_Re_A1) RQ_R_Oth_N_Cat2 =2.

IF (TH_Res=1) RQ_R_Oth_N_Cat2 =$SYSMIS.

VARIABLE LABELS RQ_R_Oth_N_Cat2 'Overeenstemming % negatief test-hertest - Respect'.

VALUE LABELS RQ_R_Oth_N_Cat2

1 'test hertest zelfde categorieen'

2 'test hertest niet in zelfde categorieen'.

FREQUENCIES RQ_R_Oth_N_Cat2.

NUMERIC RQ_A_Oth_N_Cat2 (F2.0).

COMPUTE RQ_A_Oth_N_Cat2 = -999.

IF (RQ_A_Neg_A_A1 = RQ_A_Neg_A_Re_A1) RQ_A_Oth_N_Cat2 =1.

IF (RQ_A_Neg_A_A1 <> RQ_A_Neg_A_Re_A1) RQ_A_Oth_N_Cat2 =2.

IF (TH_Res=1) RQ_A_Oth_N_Cat2 =$SYSMIS.

VARIABLE LABELS RQ_A_Oth_N_Cat2 'Overeenstemming % negatief test-hertest - Autonomie'.

VALUE LABELS RQ_A_Oth_N_Cat2

1 'test hertest zelfde categorieen'

2 'test hertest niet in zelfde categorieen'.

FREQUENCIES RQ_A_Oth_N_Cat2.

NUMERIC RQ_P_Oth_N_Cat2 (F2.0).

COMPUTE RQ_P_Oth_N_Cat2 = -999.

IF (RQ_P_Neg_A_A1 = RQ_P_Neg_A_Re_A1) RQ_P_Oth_N_Cat2 =1.

IF (RQ_P_Neg_A_A1 <> RQ_P_Neg_A_Re_A1) RQ_P_Oth_N_Cat2 =2.

IF (TH_Res=1) RQ_P_Oth_N_Cat2 =$SYSMIS.

VARIABLE LABELS RQ_P_Oth_N_Cat2 'Overeenstemming % negatief test-hertest - Privacy'.

VALUE LABELS RQ_P_Oth_N_Cat2

1 'test hertest zelfde categorieen'

2 'test hertest niet in zelfde categorieen'.

FREQUENCIES RQ_P_Oth_N_Cat2.

NUMERIC RQ_C_Oth_N_Cat2 (F2.0).

COMPUTE RQ_C_Oth_N_Cat2 = -999.

IF (RQ_C_Neg_A_A1 = RQ_C_Neg_A_Re_A1) RQ_C_Oth_N_Cat2 =1.

IF (RQ_C_Neg_A_A1 <> RQ_C_Neg_A_Re_A1) RQ_C_Oth_N_Cat2 =2.

IF (TH_Res=1) RQ_C_Oth_N_Cat2 =$SYSMIS.

VARIABLE LABELS RQ_C_Oth_N_Cat2 'Overeenstemming % negatief test-hertest - Communicatie'.

VALUE LABELS RQ_C_Oth_N_Cat2

1 'test hertest zelfde categorieen'

2 'test hertest niet in zelfde categorieen'.

FREQUENCIES RQ_C_Oth_N_Cat2.

NUMERIC RQ_T_Oth_N_Cat2 (F2.0).

COMPUTE RQ_T_Oth_N_Cat2 = -999.

IF (RQ_T_Neg_A_A1 = RQ_T_Neg_A_Re_A1) RQ_T_Oth_N_Cat2 =1.

IF (RQ_T_Neg_A_A1 <> RQ_T_Neg_A_Re_A1) RQ_T_Oth_N_Cat2 =2.

IF (TH_Res=1) RQ_T_Oth_N_Cat2 =$SYSMIS.

VARIABLE LABELS RQ_T_Oth_N_Cat2 'Overeenstemming % negatief test-hertest - Tijd tot geboden hulp'.

VALUE LABELS RQ_T_Oth_N_Cat2

1 'test hertest zelfde categorieen'

2 'test hertest niet in zelfde categorieen'.

FREQUENCIES RQ_T_Oth_N_Cat2.

NUMERIC RQ_S_Oth_N_Cat2 (F2.0).

COMPUTE RQ_S_Oth_N_Cat2 = -999.

IF (RQ_S_Neg_A_A1 = RQ_S_Neg_A_Re_A1) RQ_S_Oth_N_Cat2 =1.

IF (RQ_S_Neg_A_A1 <> RQ_S_Neg_A_Re_A1) RQ_S_Oth_N_Cat2 =2.

IF (TH_Res=1) RQ_S_Oth_N_Cat2 =$SYSMIS.

VARIABLE LABELS RQ_S_Oth_N_Cat2 'Overeenstemming % negatief test-hertest - Sociale ondersteuning'.

VALUE LABELS RQ_S_Oth_N_Cat2

1 'test hertest zelfde categorieen'

2 'test hertest niet in zelfde categorieen'.

FREQUENCIES RQ_S_Oth_N_Cat2.

NUMERIC RQ_F_Oth_N_Cat2 (F2.0).

COMPUTE RQ_F_Oth_N_Cat2 = -999.

IF (RQ_F_Neg_A_A1 = RQ_F_Neg_A_Re_A1) RQ_F_Oth_N_Cat2 =1.

IF (RQ_F_Neg_A_A1 <> RQ_F_Neg_A_Re_A1) RQ_F_Oth_N_Cat2 =2.

IF (TH_Res=1) RQ_F_Oth_N_Cat2 =$SYSMIS.

VARIABLE LABELS RQ_F_Oth_N_Cat2 'Overeenstemming % negatief test-hertest - Faciliteiten'.

VALUE LABELS RQ_F_Oth_N_Cat2

1 'test hertest zelfde categorieen'

2 'test hertest niet in zelfde categorieen'.

FREQUENCIES RQ_F_Oth_N_Cat2.

NUMERIC RQ_K_Oth_N_Cat2 (F2.0).

COMPUTE RQ_K_Oth_N_Cat2 = -999.

IF (RQ_K_Neg_A_A1 = RQ_K_Neg_A_Re_A1) RQ_K_Oth_N_Cat2 =1.

IF (RQ_K_Neg_A_A1 <> RQ_K_Neg_A_Re_A1) RQ_K_Oth_N_Cat2 =2.

IF (TH_Res=1) RQ_K_Oth_N_Cat2 =$SYSMIS.

VARIABLE LABELS RQ_K_Oth_N_Cat2 'Overeenstemming % negatief test-hertest - Keuze en continuiteit'.

VALUE LABELS RQ_K_Oth_N_Cat2

1 'test hertest zelfde categorieen'

2 'test hertest niet in zelfde categorieen'.

FREQUENCIES RQ_K_Oth_N_Cat2.

NUMERIC RQ_PS_Oth_N_Cat2 (F2.0).

COMPUTE RQ_PS_Oth_N_Cat2 = -999.

IF (RQ_PS_Neg_A_A1 = RQ_PS_Neg_A_Re_A1) RQ_PS_Oth_N_Cat2 =1.

IF (RQ_PS_Neg_A_A1 <> RQ_PS_Neg_A_Re_A1) RQ_PS_Oth_N_Cat2 =2.

IF (TH_Res=1) RQ_PS_Oth_N_Cat2 =$SYSMIS.

VARIABLE LABELS RQ_PS_Oth_N_Cat2 'Overeenstemming % negatief test-hertest - Personal score'.

VALUE LABELS RQ_PS_Oth_N_Cat2

1 'test hertest zelfde categorieen'

2 'test hertest niet in zelfde categorieen'.

FREQUENCIES RQ_PS_Oth_N_Cat2.

NUMERIC RQ_ST_Oth_N_Cat2 (F2.0).

COMPUTE RQ_ST_Oth_N_Cat2 = -999.

IF (RQ_ST_Neg_A_A1 = RQ_ST_Neg_A_Re_A1) RQ_ST_Oth_N_Cat2 =1.

IF (RQ_ST_Neg_A_A1 <> RQ_ST_Neg_A_Re_A1) RQ_ST_Oth_N_Cat2 =2.

IF (TH_Res=1) RQ_ST_Oth_N_Cat2 =$SYSMIS.

VARIABLE LABELS RQ_ST_Oth_N_Cat2 'Overeenstemming % negatief test-hertest - setting score'.

VALUE LABELS RQ_ST_Oth_N_Cat2

1 'test hertest zelfde categorieen'

2 'test hertest niet in zelfde categorieen'.

FREQUENCIES RQ_ST_Oth_N_Cat2.

NUMERIC RQ_EvT_Oth_N_Cat2 (F2.0).

COMPUTE RQ_EvT_Oth_N_Cat2 = -999.

IF (RQ_EVT_Neg_A_A1 = RQ_EVT_Neg_A_Re_A1) RQ_EvT_Oth_N_Cat2 =1.

IF (RQ_EVT_Neg_A_A1 <> RQ_EVT_Neg_A_Re_A1) RQ_EvT_Oth_N_Cat2 =2.

IF (TH_Res=1) RQ_EvT_Oth_N_Cat2 =$SYSMIS.

VARIABLE LABELS RQ_EvT_Oth_N_Cat2 'Overeenstemming % negatief test-hertest - Overall score'.

VALUE LABELS RQ_EvT_Oth_N_Cat2

1 'test hertest zelfde categorieen'

2 'test hertest niet in zelfde categorieen'.

FREQUENCIES RQ_EvT_Oth_N_Cat2.

* STAP 4: % Negatief, kappa.

*ALTERNATIEF 2: ALLEEN MEEGENOMEN ALS IN MEEST BELANGRIJKE DOMEIN NOOIT OF SOMS.

*Respect.

SORT CASES BY F_TH_Res.

FILTER BY F_TH_Res.

FREQUENCIES RQ_R_Neg_A_A2 RQ_R_Neg_A_Re_A2.

FILTER OFF.

CROSSTABS TABLES = RQ_R_Neg_A_A2 BY RQ_R_Neg_A_Re_A2

/CELLS COUNT ROW EXPECTED COLUMN RESID

/STATISTICS KAPPA.

*Autonomie.

SORT CASES BY F_TH_Res.

FILTER BY F_TH_Res.

FREQUENCIES RQ_A_Neg_A_A2 RQ_A_Neg_A_Re_A2.

FILTER OFF.

CROSSTABS TABLES = RQ_A_Neg_A_A2 BY RQ_A_Neg_A_Re_A2

/CELLS COUNT ROW EXPECTED COLUMN RESID

/STATISTICS KAPPA.

*Privacy.

SORT CASES BY F_TH_Res.

FILTER BY F_TH_Res.

FREQUENCIES RQ_P_Neg_A_A2 RQ_P_Neg_A_Re_A2.

FILTER OFF.

CROSSTABS TABLES = RQ_P_Neg_A_A2 BY RQ_P_Neg_A_Re_A2

/CELLS COUNT ROW EXPECTED COLUMN RESID

/STATISTICS KAPPA.

*Communicatie.

SORT CASES BY F_TH_Res.

FILTER BY F_TH_Res.

FREQUENCIES RQ_C_Neg_A_A2 RQ_C_Neg_A_Re_A2.

FILTER OFF.

CROSSTABS TABLES = RQ_C_Neg_A_A2 BY RQ_C_Neg_A_Re_A2

/CELLS COUNT ROW EXPECTED COLUMN RESID

/STATISTICS KAPPA.

*Tijd tot geboden hulp.

SORT CASES BY F_TH_Res.

FILTER BY F_TH_Res.

FREQUENCIES RQ_T_Neg_A_A2 RQ_T_Neg_A_Re_A2.

FILTER OFF.

CROSSTABS TABLES = RQ_T_Neg_A_A2 BY RQ_T_Neg_A_Re_A2

/CELLS COUNT ROW EXPECTED COLUMN RESID

/STATISTICS KAPPA.

*Sociale ondersteuning.

SORT CASES BY F_TH_Res.

FILTER BY F_TH_Res.

FREQUENCIES RQ_S_Neg_A_A2 RQ_S_Neg_A_Re_A2.

FILTER OFF.

CROSSTABS TABLES = RQ_S_Neg_A_A2 BY RQ_S_Neg_A_Re_A2

/CELLS COUNT ROW EXPECTED COLUMN RESID

/STATISTICS KAPPA.

*Faciliteiten.

SORT CASES BY F_TH_Res.

FILTER BY F_TH_Res.

FREQUENCIES RQ_F_Neg_A_A2 RQ_F_Neg_A_Re_A2.

FILTER OFF.

CROSSTABS TABLES = RQ_F_Neg_A_A2 BY RQ_F_Neg_A_Re_A2

/CELLS COUNT ROW EXPECTED COLUMN RESID

/STATISTICS KAPPA.

*Keuze en continuiteit.

SORT CASES BY F_TH_Res.

FILTER BY F_TH_Res.

FREQUENCIES RQ_K_Neg_A_A2 RQ_K_Neg_A_Re_A2.

FILTER OFF.

CROSSTABS TABLES = RQ_K_Neg_A_A2 BY RQ_K_Neg_A_Re_A2

/CELLS COUNT ROW EXPECTED COLUMN RESID

/STATISTICS KAPPA.

*Personal.

SORT CASES BY F_TH_Res.

FILTER BY F_TH_Res.

FREQUENCIES RQ_PS_Neg_A_A2 RQ_PS_Neg_A_RE_A2.

FILTER OFF.

CROSSTABS TABLES = RQ_PS_Neg_A_A2 BY RQ_PS_Neg_A_RE_A2

/CELLS COUNT ROW EXPECTED COLUMN RESID

/STATISTICS KAPPA.

*Setting.

SORT CASES BY F_TH_Res.

FILTER BY F_TH_Res.

FREQUENCIES RQ_ST_Neg_A_A2 RQ_ST_Neg_A_RE_A2.

FILTER OFF.

CROSSTABS TABLES = RQ_ST_Neg_A_A2 BY RQ_ST_Neg_A_RE_A2

/CELLS COUNT ROW EXPECTED COLUMN RESID

/STATISTICS KAPPA.

*overall score.

SORT CASES BY F_TH_Res.

FILTER BY F_TH_Res.

FREQUENCIES RQ_EvT_Neg_A_A2 RQ_EvT_Neg_A_RE_A2.

FILTER OFF.

CROSSTABS TABLES = RQ_EvT_Neg_A_A2 BY RQ_EvT_Neg_A_RE_A2

/CELLS COUNT ROW EXPECTED COLUMN RESID

/STATISTICS KAPPA.

* STAP 5: % overeenstemming in % negatief .

NUMERIC RQ_R_Oth_N_A2 (F2.0).

COMPUTE RQ_R_Oth_N_A2 = -999.

IF (RQ_R_Neg_A_A2 = RQ_R_Neg_A_Re_A2) RQ_R_Oth_N_A2 =1.

IF (RQ_R_Neg_A_A2 <> RQ_R_Neg_A_Re_A2) RQ_R_Oth_N_A2 =2.

IF (TH_Res=1) RQ_R_Oth_N_A2 =$SYSMIS.

VARIABLE LABELS RQ_R_Oth_N_A2 'Overeenstemming % negatief test-hertest - Respect'.

VALUE LABELS RQ_R_Oth_N_A2

1 'test hertest zelfde categorieen'

2 'test hertest niet in zelfde categorieen'.

FREQUENCIES RQ_R_Oth_N_A2.

NUMERIC RQ_A_Oth_N_A2 (F2.0).

COMPUTE RQ_A_Oth_N_A2 = -999.

IF (RQ_A_Neg_A_A2 = RQ_A_Neg_A_Re_A2) RQ_A_Oth_N_A2 =1.

IF (RQ_A_Neg_A_A2 <> RQ_A_Neg_A_Re_A2) RQ_A_Oth_N_A2 =2.

IF (TH_Res=1) RQ_A_Oth_N_A2 =$SYSMIS.

VARIABLE LABELS RQ_A_Oth_N_A2 'Overeenstemming % negatief test-hertest - Autonomie'.

VALUE LABELS RQ_A_Oth_N_A2

1 'test hertest zelfde categorieen'

2 'test hertest niet in zelfde categorieen'.

FREQUENCIES RQ_A_Oth_N_A2.

NUMERIC RQ_P_Oth_N_A2 (F2.0).

COMPUTE RQ_P_Oth_N_A2 = -999.

IF (RQ_P_Neg_A_A2 = RQ_P_Neg_A_Re_A2) RQ_P_Oth_N_A2 =1.

IF (RQ_P_Neg_A_A2 <> RQ_P_Neg_A_Re_A2) RQ_P_Oth_N_A2 =2.

IF (TH_Res=1) RQ_P_Oth_N_A2 =$SYSMIS.

VARIABLE LABELS RQ_P_Oth_N_A2 'Overeenstemming % negatief test-hertest - Privacy'.

VALUE LABELS RQ_P_Oth_N_A2

1 'test hertest zelfde categorieen'

2 'test hertest niet in zelfde categorieen'.

FREQUENCIES RQ_P_Oth_N_A2.

NUMERIC RQ_C_Oth_N_A2 (F2.0).

COMPUTE RQ_C_Oth_N_A2 = -999.

IF (RQ_C_Neg_A_A2 = RQ_C_Neg_A_Re_A2) RQ_C_Oth_N_A2 =1.

IF (RQ_C_Neg_A_A2 <> RQ_C_Neg_A_Re_A2) RQ_C_Oth_N_A2 =2.

IF (TH_Res=1) RQ_C_Oth_N_A2 =$SYSMIS.

VARIABLE LABELS RQ_C_Oth_N_A2 'Overeenstemming % negatief test-hertest - Communicatie'.

VALUE LABELS RQ_C_Oth_N_A2

1 'test hertest zelfde categorieen'

2 'test hertest niet in zelfde categorieen'.

FREQUENCIES RQ_C_Oth_N_A2.

NUMERIC RQ_T_Oth_N_A2 (F2.0).

COMPUTE RQ_T_Oth_N_A2 = -999.

IF (RQ_T_Neg_A_A2 = RQ_T_Neg_A_Re_A2) RQ_T_Oth_N_A2 =1.

IF (RQ_T_Neg_A_A2 <> RQ_T_Neg_A_Re_A2) RQ_T_Oth_N_A2 =2.

IF (TH_Res=1) RQ_T_Oth_N_A2 =$SYSMIS.

VARIABLE LABELS RQ_T_Oth_N_A2 'Overeenstemming % negatief test-hertest - Tijd tot geboden hulp'.

VALUE LABELS RQ_T_Oth_N_A2

1 'test hertest zelfde categorieen'

2 'test hertest niet in zelfde categorieen'.

FREQUENCIES RQ_T_Oth_N_A2.

NUMERIC RQ_S_Oth_N_A2 (F2.0).

COMPUTE RQ_S_Oth_N_A2 = -999.

IF (RQ_S_Neg_A_A2 = RQ_S_Neg_A_Re_A2) RQ_S_Oth_N_A2 =1.

IF (RQ_S_Neg_A_A2 <> RQ_S_Neg_A_Re_A2) RQ_S_Oth_N_A2 =2.

IF (TH_Res=1) RQ_S_Oth_N_A2 =$SYSMIS.

VARIABLE LABELS RQ_S_Oth_N_A2 'Overeenstemming % negatief test-hertest - Sociale ondersteuning'.

VALUE LABELS RQ_S_Oth_N_A2

1 'test hertest zelfde categorieen'

2 'test hertest niet in zelfde categorieen'.

FREQUENCIES RQ_S_Oth_N_A2.

NUMERIC RQ_F_Oth_N_A2 (F2.0).

COMPUTE RQ_F_Oth_N_A2 = -999.

IF (RQ_F_Neg_A_A2 = RQ_F_Neg_A_Re_A2) RQ_F_Oth_N_A2 =1.

IF (RQ_F_Neg_A_A2 <> RQ_F_Neg_A_Re_A2) RQ_F_Oth_N_A2 =2.

IF (TH_Res=1) RQ_F_Oth_N_A2 =$SYSMIS.

VARIABLE LABELS RQ_F_Oth_N_A2 'Overeenstemming % negatief test-hertest - Faciliteiten'.

VALUE LABELS RQ_F_Oth_N_A2

1 'test hertest zelfde categorieen'

2 'test hertest niet in zelfde categorieen'.

FREQUENCIES RQ_F_Oth_N_A2.

NUMERIC RQ_K_Oth_N_A2 (F2.0).

COMPUTE RQ_K_Oth_N_A2 = -999.

IF (RQ_K_Neg_A_A2 = RQ_K_Neg_A_Re_A2) RQ_K_Oth_N_A2 =1.

IF (RQ_K_Neg_A_A2 <> RQ_K_Neg_A_Re_A2) RQ_K_Oth_N_A2 =2.

IF (TH_Res=1) RQ_K_Oth_N_A2 =$SYSMIS.

VARIABLE LABELS RQ_K_Oth_N_A2 'Overeenstemming % negatief test-hertest - Keuze en continuiteit'.

VALUE LABELS RQ_K_Oth_N_A2

1 'test hertest zelfde categorieen'

2 'test hertest niet in zelfde categorieen'.

FREQUENCIES RQ_K_Oth_N_A2.

NUMERIC RQ_PS_Oth_N_A2 (F2.0).

COMPUTE RQ_PS_Oth_N_A2 = -999.

IF (RQ_PS_Neg_A_A2 = RQ_PS_Neg_A_Re_A2) RQ_PS_Oth_N_A2 =1.

IF (RQ_PS_Neg_A_A2 <> RQ_PS_Neg_A_Re_A2) RQ_PS_Oth_N_A2 =2.

IF (TH_Res=1) RQ_PS_Oth_N_A2 =$SYSMIS.

VARIABLE LABELS RQ_PS_Oth_N_A2 'Overeenstemming % negatief test-hertest - Personal score'.

VALUE LABELS RQ_PS_Oth_N_A2

1 'test hertest zelfde categorieen'

2 'test hertest niet in zelfde categorieen'.

FREQUENCIES RQ_PS_Oth_N_A2.

NUMERIC RQ_ST_Oth_N_A2 (F2.0).

COMPUTE RQ_ST_Oth_N_A2 = -999.

IF (RQ_ST_Neg_A_A2 = RQ_ST_Neg_A_Re_A2) RQ_ST_Oth_N_A2 =1.

IF (RQ_ST_Neg_A_A2 <> RQ_ST_Neg_A_Re_A2) RQ_ST_Oth_N_A2 =2.

IF (TH_Res=1) RQ_ST_Oth_N_A2 =$SYSMIS.

VARIABLE LABELS RQ_ST_Oth_N_A2 'Overeenstemming % negatief test-hertest - setting score'.

VALUE LABELS RQ_ST_Oth_N_A2

1 'test hertest zelfde categorieen'

2 'test hertest niet in zelfde categorieen'.

FREQUENCIES RQ_ST_Oth_N_A2.

NUMERIC RQ_EvT_Oth_N_A2 (F2.0).

COMPUTE RQ_EvT_Oth_N_A2 = -999.

IF (RQ_EVT_Neg_A_A2 = RQ_EVT_Neg_A_Re_A2) RQ_EvT_Oth_N_A2 =1.

IF (RQ_EVT_Neg_A_A2 <> RQ_EVT_Neg_A_Re_A2) RQ_EvT_Oth_N_A2 =2.

IF (TH_Res=1) RQ_EvT_Oth_N_A2 =$SYSMIS.

VARIABLE LABELS RQ_EvT_Oth_N_A2 'Overeenstemming % negatief test-hertest - Overall score'.

VALUE LABELS RQ_EvT_Oth_N_A2

1 'test hertest zelfde categorieen'

2 'test hertest niet in zelfde categorieen'.

FREQUENCIES RQ_EvT_Oth_N_A2.

* STAP 4: % Negatief, kappa.

*ALTERNATIEF 3: NOOIT IN ERGENS EN SOMS ALS IN MEEST BELANGRIJKE DOMEIN.

*Respect.

SORT CASES BY F_TH_Res.

FILTER BY F_TH_Res.

FREQUENCIES RQ_R_Neg_A_A3 RQ_R_Neg_A_Re_A3.

FILTER OFF.

CROSSTABS TABLES = RQ_R_Neg_A_A3 BY RQ_R_Neg_A_Re_A3

/CELLS COUNT ROW EXPECTED COLUMN RESID

/STATISTICS KAPPA.

*Autonomie.

SORT CASES BY F_TH_Res.

FILTER BY F_TH_Res.

FREQUENCIES RQ_A_Neg_A_A3 RQ_A_Neg_A_Re_A3.

FILTER OFF.

CROSSTABS TABLES = RQ_A_Neg_A_A3 BY RQ_A_Neg_A_Re_A3

/CELLS COUNT ROW EXPECTED COLUMN RESID

/STATISTICS KAPPA.

*Privacy.

SORT CASES BY F_TH_Res.

FILTER BY F_TH_Res.

FREQUENCIES RQ_P_Neg_A_A3 RQ_P_Neg_A_Re_A3.

FILTER OFF.

CROSSTABS TABLES = RQ_P_Neg_A_A3 BY RQ_P_Neg_A_Re_A3

/CELLS COUNT ROW EXPECTED COLUMN RESID

/STATISTICS KAPPA.

*Communicatie.

SORT CASES BY F_TH_Res.

FILTER BY F_TH_Res.

FREQUENCIES RQ_C_Neg_A_A3 RQ_C_Neg_A_Re_A3.

FILTER OFF.

CROSSTABS TABLES = RQ_C_Neg_A_A3 BY RQ_C_Neg_A_Re_A3

/CELLS COUNT ROW EXPECTED COLUMN RESID

/STATISTICS KAPPA.

*Tijd tot geboden hulp.

SORT CASES BY F_TH_Res.

FILTER BY F_TH_Res.

FREQUENCIES RQ_T_Neg_A_A3 RQ_T_Neg_A_Re_A3.

FILTER OFF.

CROSSTABS TABLES = RQ_T_Neg_A_A3 BY RQ_T_Neg_A_Re_A3

/CELLS COUNT ROW EXPECTED COLUMN RESID

/STATISTICS KAPPA.

*Sociale ondersteuning.

SORT CASES BY F_TH_Res.

FILTER BY F_TH_Res.

FREQUENCIES RQ_S_Neg_A_A3 RQ_S_Neg_A_Re_A3.

FILTER OFF.

CROSSTABS TABLES = RQ_S_Neg_A_A3 BY RQ_S_Neg_A_Re_A3

/CELLS COUNT ROW EXPECTED COLUMN RESID

/STATISTICS KAPPA.

*Faciliteiten.

SORT CASES BY F_TH_Res.

FILTER BY F_TH_Res.

FREQUENCIES RQ_F_Neg_A_A3 RQ_F_Neg_A_Re_A3.

FILTER OFF.

CROSSTABS TABLES = RQ_F_Neg_A_A3 BY RQ_F_Neg_A_Re_A3

/CELLS COUNT ROW EXPECTED COLUMN RESID

/STATISTICS KAPPA.

*Keuze en continuiteit.

SORT CASES BY F_TH_Res.

FILTER BY F_TH_Res.

FREQUENCIES RQ_K_Neg_A_A3 RQ_K_Neg_A_Re_A3.

FILTER OFF.

CROSSTABS TABLES = RQ_K_Neg_A_A3 BY RQ_K_Neg_A_Re_A3

/CELLS COUNT ROW EXPECTED COLUMN RESID

/STATISTICS KAPPA.

*Personal.

SORT CASES BY F_TH_Res.

FILTER BY F_TH_Res.

FREQUENCIES RQ_PS_Neg_A_A3 RQ_PS_Neg_A_RE_A3.

FILTER OFF.

CROSSTABS TABLES = RQ_PS_Neg_A_A3 BY RQ_PS_Neg_A_RE_A3

/CELLS COUNT ROW EXPECTED COLUMN RESID

/STATISTICS KAPPA.

*Setting.

SORT CASES BY F_TH_Res.

FILTER BY F_TH_Res.

FREQUENCIES RQ_ST_Neg_A_A3 RQ_ST_Neg_A_RE_A3.

FILTER OFF.

CROSSTABS TABLES = RQ_ST_Neg_A_A3 BY RQ_ST_Neg_A_RE_A3

/CELLS COUNT ROW EXPECTED COLUMN RESID

/STATISTICS KAPPA.

*overall score.

SORT CASES BY F_TH_Res.

FILTER BY F_TH_Res.

FREQUENCIES RQ_EvT_Neg_A_A3 RQ_EvT_Neg_A_RE_A3.

CROSSTABS TABLES = RQ_EvT_Neg_A_A3 BY RQ_EvT_Neg_A_RE_A3

/CELLS COUNT ROW EXPECTED COLUMN RESID

/STATISTICS KAPPA.

FILTER OFF.

* STAP 5: % overeenstemming in % negatief .

SORT CASES BY F_TH_Res.

FILTER BY F_TH_Res.

NUMERIC RQ_R_Oth_N_A3 (F2.0).

COMPUTE RQ_R_Oth_N_A3 = -999.

IF (RQ_R_Neg_A_A3 = RQ_R_Neg_A_Re_A3) RQ_R_Oth_N_A3 =1.

IF (RQ_R_Neg_A_A3 <> RQ_R_Neg_A_Re_A3) RQ_R_Oth_N_A3 =2.

IF (TH_Res=1) RQ_R_Oth_N_A3 =$SYSMIS.

VARIABLE LABELS RQ_R_Oth_N_A3 'Overeenstemming % negatief test-hertest - Respect'.

VALUE LABELS RQ_R_Oth_N_A3

1 'test hertest zelfde categorieen'

2 'test hertest niet in zelfde categorieen'.

FREQUENCIES RQ_R_Oth_N_A3.

NUMERIC RQ_A_Oth_N_A3 (F2.0).

COMPUTE RQ_A_Oth_N_A3 = -999.

IF (RQ_A_Neg_A_A3 = RQ_A_Neg_A_Re_A3) RQ_A_Oth_N_A3 =1.

IF (RQ_A_Neg_A_A3 <> RQ_A_Neg_A_Re_A3) RQ_A_Oth_N_A3 =2.

IF (TH_Res=1) RQ_A_Oth_N_A3 =$SYSMIS.

VARIABLE LABELS RQ_A_Oth_N_A3 'Overeenstemming % negatief test-hertest - Autonomie'.

VALUE LABELS RQ_A_Oth_N_A3

1 'test hertest zelfde categorieen'

2 'test hertest niet in zelfde categorieen'.

FREQUENCIES RQ_A_Oth_N_A3.

NUMERIC RQ_P_Oth_N_A3 (F2.0).

COMPUTE RQ_P_Oth_N_A3 = -999.

IF (RQ_P_Neg_A_A3 = RQ_P_Neg_A_Re_A3) RQ_P_Oth_N_A3 =1.

IF (RQ_P_Neg_A_A3 <> RQ_P_Neg_A_Re_A3) RQ_P_Oth_N_A3 =2.

IF (TH_Res=1) RQ_P_Oth_N_A3 =$SYSMIS.

VARIABLE LABELS RQ_P_Oth_N_A3 'Overeenstemming % negatief test-hertest - Privacy'.

VALUE LABELS RQ_P_Oth_N_A3

1 'test hertest zelfde categorieen'

2 'test hertest niet in zelfde categorieen'.

FREQUENCIES RQ_P_Oth_N_A3.

NUMERIC RQ_C_Oth_N_A3 (F2.0).

COMPUTE RQ_C_Oth_N_A3 = -999.

IF (RQ_C_Neg_A_A3 = RQ_C_Neg_A_Re_A3) RQ_C_Oth_N_A3 =1.

IF (RQ_C_Neg_A_A3 <> RQ_C_Neg_A_Re_A3) RQ_C_Oth_N_A3 =2.

IF (TH_Res=1) RQ_C_Oth_N_A3 =$SYSMIS.

VARIABLE LABELS RQ_C_Oth_N_A3 'Overeenstemming % negatief test-hertest - Communicatie'.

VALUE LABELS RQ_C_Oth_N_A3

1 'test hertest zelfde categorieen'

2 'test hertest niet in zelfde categorieen'.

FREQUENCIES RQ_C_Oth_N_A3.

NUMERIC RQ_T_Oth_N_A3 (F2.0).

COMPUTE RQ_T_Oth_N_A3 = -999.

IF (RQ_T_Neg_A_A3 = RQ_T_Neg_A_Re_A3) RQ_T_Oth_N_A3 =1.

IF (RQ_T_Neg_A_A3 <> RQ_T_Neg_A_Re_A3) RQ_T_Oth_N_A3 =2.

IF (TH_Res=1) RQ_T_Oth_N_A3 =$SYSMIS.

VARIABLE LABELS RQ_T_Oth_N_A3 'Overeenstemming % negatief test-hertest - Tijd tot geboden hulp'.

VALUE LABELS RQ_T_Oth_N_A3

1 'test hertest zelfde categorieen'

2 'test hertest niet in zelfde categorieen'.

FREQUENCIES RQ_T_Oth_N_A3.

NUMERIC RQ_S_Oth_N_A3 (F2.0).

COMPUTE RQ_S_Oth_N_A3 = -999.

IF (RQ_S_Neg_A_A3 = RQ_S_Neg_A_Re_A3) RQ_S_Oth_N_A3 =1.

IF (RQ_S_Neg_A_A3 <> RQ_S_Neg_A_Re_A3) RQ_S_Oth_N_A3 =2.

IF (TH_Res=1) RQ_S_Oth_N_A3 =$SYSMIS.

VARIABLE LABELS RQ_S_Oth_N_A3 'Overeenstemming % negatief test-hertest - Sociale ondersteuning'.

VALUE LABELS RQ_S_Oth_N_A3

1 'test hertest zelfde categorieen'

2 'test hertest niet in zelfde categorieen'.

FREQUENCIES RQ_S_Oth_N_A3.

NUMERIC RQ_F_Oth_N_A3 (F2.0).

COMPUTE RQ_F_Oth_N_A3 = -999.

IF (RQ_F_Neg_A_A3 = RQ_F_Neg_A_Re_A3) RQ_F_Oth_N_A3 =1.

IF (RQ_F_Neg_A_A3 <> RQ_F_Neg_A_Re_A3) RQ_F_Oth_N_A3 =2.

IF (TH_Res=1) RQ_F_Oth_N_A3 =$SYSMIS.

VARIABLE LABELS RQ_F_Oth_N_A3 'Overeenstemming % negatief test-hertest - Faciliteiten'.

VALUE LABELS RQ_F_Oth_N_A3

1 'test hertest zelfde categorieen'

2 'test hertest niet in zelfde categorieen'.

FREQUENCIES RQ_F_Oth_N_A3.

NUMERIC RQ_K_Oth_N_A3 (F2.0).

COMPUTE RQ_K_Oth_N_A3 = -999.

IF (RQ_K_Neg_A_A3 = RQ_K_Neg_A_Re_A3) RQ_K_Oth_N_A3 =1.

IF (RQ_K_Neg_A_A3 <> RQ_K_Neg_A_Re_A3) RQ_K_Oth_N_A3 =2.

IF (TH_Res=1) RQ_K_Oth_N_A3 =$SYSMIS.

VARIABLE LABELS RQ_K_Oth_N_A3 'Overeenstemming % negatief test-hertest - Keuze en continuiteit'.

VALUE LABELS RQ_K_Oth_N_A3

1 'test hertest zelfde categorieen'

2 'test hertest niet in zelfde categorieen'.

FREQUENCIES RQ_K_Oth_N_A3.

NUMERIC RQ_PS_Oth_N_A3 (F2.0).

COMPUTE RQ_PS_Oth_N_A3 = -999.

IF (RQ_PS_Neg_A_A3 = RQ_PS_Neg_A_Re_A3) RQ_PS_Oth_N_A3 =1.

IF (RQ_PS_Neg_A_A3 <> RQ_PS_Neg_A_Re_A3) RQ_PS_Oth_N_A3 =2.

IF (TH_Res=1) RQ_PS_Oth_N_A3 =$SYSMIS.

VARIABLE LABELS RQ_PS_Oth_N_A3 'Overeenstemming % negatief test-hertest - Personal score'.

VALUE LABELS RQ_PS_Oth_N_A3

1 'test hertest zelfde categorieen'

2 'test hertest niet in zelfde categorieen'.

FREQUENCIES RQ_PS_Oth_N_A3.

NUMERIC RQ_ST_Oth_N_A3 (F2.0).

COMPUTE RQ_ST_Oth_N_A3 = -999.

IF (RQ_ST_Neg_A_A3 = RQ_ST_Neg_A_Re_A3) RQ_ST_Oth_N_A3 =1.

IF (RQ_ST_Neg_A_A3 <> RQ_ST_Neg_A_Re_A3) RQ_ST_Oth_N_A3 =2.

IF (TH_Res=1) RQ_ST_Oth_N_A3 =$SYSMIS.

VARIABLE LABELS RQ_ST_Oth_N_A3 'Overeenstemming % negatief test-hertest - setting score'.

VALUE LABELS RQ_ST_Oth_N_A3

1 'test hertest zelfde categorieen'

2 'test hertest niet in zelfde categorieen'.

FREQUENCIES RQ_ST_Oth_N_A3.

NUMERIC RQ_EvT_Oth_N_A3 (F2.0).

COMPUTE RQ_EvT_Oth_N_A3 = -999.

IF (RQ_EVT_Neg_A_A3 = RQ_EVT_Neg_A_Re_A3) RQ_EvT_Oth_N_A3 =1.

IF (RQ_EVT_Neg_A_A3 <> RQ_EVT_Neg_A_Re_A3) RQ_EvT_Oth_N_A3 =2.

IF (TH_Res=1) RQ_EvT_Oth_N_A3 =$SYSMIS.

VARIABLE LABELS RQ_EvT_Oth_N_A3 'Overeenstemming % negatief test-hertest - Overall score'.

VALUE LABELS RQ_EvT_Oth_N_A3

1 'test hertest zelfde categorieen'

2 'test hertest niet in zelfde categorieen'.

FREQUENCIES RQ_EvT_Oth_N_A3.

FILTER OFF.

# MINIMALLY IMPORTANT DIFFERENCE

* STAP 1: Gemiddelde domein en totaal scores voor 5 groepen overall rating tegen elkaar uitzetten.

FREQUENCIES OV_All_M_Cat5.

SORT CASES OV_All_M_Cat5 (A).

SPLIT FILE BY OV_All_M_Cat5.

FREQUENCIES VARIABLES=RQ_R_Dom_A RQ_A_Dom_A RQ_P_Dom_A RQ_C_Dom_A RQ_T_Dom_A RQ_S_Dom_A RQ_F_Dom_A RQ_K_Dom_A RQ_PS_Dom_A RQ_ST_Dom_A RQ_EvT_A

/STATISTICS=MEAN STDDEV MEDIAN

/NTILES 4

/ORDER=ANALYSIS.

SPLIT FILE OFF.

* STAP 2: Berekenen standard error of the mean.

DESCRIPTIVES VARIABLES=RQ_R_Dom_A RQ_A_Dom_A RQ_P_Dom_A RQ_C_Dom_A RQ_T_Dom_A RQ_S_Dom_A RQ_F_Dom_A

RQ_K_Dom_A RQ_PS_Dom_A RQ_ST_Dom_A RQ_EvT_A

/STATISTICS=MEAN STDDEV MIN MAX SEMEAN.

* STAP 4: % Negatief domein voor 5 groepen overall rating tegen elkaar uitzetten.

SORT CASES OV_All_M_Cat5 (A).

SPLIT FILE BY OV_All_M_Cat5.

FREQUENCIES VARIABLES=

RQ_R_Neg_A_A3

RQ_A_Neg_A_A3

RQ_P_Neg_A_A3

RQ_C_Neg_A_A3

RQ_T_Neg_A_A3

RQ_S_Neg_A_A3

RQ_F_Neg_A_A3

RQ_K_Neg_A_A3

RQ_PS_Neg_A_A3

RQ_ST_Neg_A_A3

RQ_EVT_Neg_A_A3

/STATISTICS=MEAN STDDEV.

SPLIT FILE OFF.

# DISCRIMINATIVE POWER

* PROJECT: POSTNATALE REPROQ - BV.

* DOEL: Known-groups.

* DATUM: 24-06-2014

* AFSPRAKEN:

* Commando's in hoofdletters.

* Elke var begint met hoofdletter.

* Elke omschrijving begint met hoofdletter.

* VERWERKINGSSTAPPEN:

* STAP 1: Berekenen gem, mediaan en % negatief voor verschillende known-groups.

* STAP 2: .

* STAP 3: .

* STAP 4:

* STAP 5:

*BIJZONDERHEDEN.

*Groepen:.

*pathway.

* caesarean.

* knowning professional supervising delivery.

* ubranasation.

* office hours.

* travel time.

FREQUENCIES Zorgproces_M_Cat4 ZB_Kjn_M_Cat3 OV_Kza_M_Cat2 ZB_Urb_M ZB_Kan_M_Cat2 ZB_Ret_M_Cat2 VSV_GR_M.

*PATHWAY/RISK.

SORT CASES BY Zorgproces_M_Cat4 (A).

SPLIT FILE BY Zorgproces_M_Cat4.

FREQUENCIES VARIABLES=RQ_R_Dom_A RQ_A_Dom_A RQ_P_Dom_A RQ_C_Dom_A RQ_T_Dom_A RQ_S_Dom_A RQ_F_Dom_A RQ_K_Dom_A RQ_PS_Dom_A RQ_ST_Dom_A RQ_EvT_A

/STATISTICS=MEAN MEDIAN

/NTILES 4

/ORDER=ANALYSIS.

FREQUENCIES RQ_R_Neg_A_A3 RQ_A_Neg_A_A3 RQ_P_Neg_A_A3 RQ_C_Neg_A_A3

RQ_T_Neg_A_A3 RQ_S_Neg_A_A3 RQ_F_Neg_A_A3 RQ_K_Neg_A_A3

RQ_PS_Neg_A_A3 RQ_ST_Neg_A_A3 RQ_EVT_Neg_A_A3.

SPLIT FILE OFF.

NPTESTS

/INDEPENDENT TEST (RQ_R_Dom_A RQ_A_Dom_A RQ_P_Dom_A RQ_C_Dom_A RQ_T_Dom_A RQ_S_Dom_A RQ_F_Dom_A RQ_K_Dom_A RQ_PS_Dom_A RQ_ST_Dom_A RQ_EvT_A) GROUP (Zorgproces_M_Cat4)

/MISSING SCOPE=ANALYSIS USERMISSING=EXCLUDE

/CRITERIA ALPHA=0.05 CILEVEL=95.

CROSSTABS

/TABLES=Zorgproces_M_Cat4 BY RQ_R_Neg_A_A3 RQ_A_Neg_A_A3 RQ_P_Neg_A_A3 RQ_C_Neg_A_A3

RQ_T_Neg_A_A3 RQ_S_Neg_A_A3 RQ_F_Neg_A_A3 RQ_K_Neg_A_A3

RQ_PS_Neg_A_A3 RQ_ST_Neg_A_A3 RQ_EVT_Neg_A_A3

/FORMAT=AVALUE TABLES

/STATISTICS=CHISQ PHI

/CELLS=COUNT

/COUNT ROUND CELL.

*KEIZERSNEE.

SORT CASES BY ZB_Kjn_M_Cat2 (A).

SPLIT FILE BY ZB_Kjn_M_Cat2.

FREQUENCIES VARIABLES=RQ_R_Dom_A RQ_A_Dom_A RQ_P_Dom_A RQ_C_Dom_A RQ_T_Dom_A RQ_S_Dom_A RQ_F_Dom_A RQ_K_Dom_A RQ_PS_Dom RQ_ST_Dom RQ_EvT_A

/STATISTICS=MEAN MEDIAN

/NTILES 4

/ORDER=ANALYSIS.

FREQUENCIES RQ_R_Neg_A_A3 RQ_A_Neg_A_A3 RQ_P_Neg_A_A3 RQ_C_Neg_A_A3

RQ_T_Neg_A_A3 RQ_S_Neg_A_A3 RQ_F_Neg_A_A3 RQ_K_Neg_A_A3

RQ_PS_Neg_A_A3 RQ_ST_Neg_A_A3 RQ_EVT_Neg_A_A3.

SPLIT FILE OFF.

NUMERIC F_Kei (F2.0).

COMPUTE F_Kei = $SYSMIS.

IF (ZB_Kjn_S_Cat3=2 OR ZB_Kjn_S_Cat3=3) F_Kei = 1.

VARIABLE LABELS F_Kei 'Filter vrouwen die zijn bevallen met een keizersnee'.

VALUE LABELS F_Kei

1 'vrouwen die zijn bevallen dmw een keizersnee'.

FREQUENCIES F_Kei.

SORT CASES BY F_Kei (A).

FILTER BY F_Kei.

NPTESTS

/INDEPENDENT TEST (RQ_R_Dom_A RQ_A_Dom_A RQ_P_Dom_A RQ_C_Dom_A RQ_T_Dom_A RQ_S_Dom_A RQ_F_Dom_A RQ_K_Dom_A RQ_PS_Dom_A RQ_ST_Dom_A RQ_EvT_A) GROUP (ZB_Kjn_M_Cat2)

/MISSING SCOPE=ANALYSIS USERMISSING=EXCLUDE

/CRITERIA ALPHA=0.05 CILEVEL=95.

CROSSTABS

/TABLES=ZB_Kjn_M_Cat2 BY RQ_R_Neg_A_A3 RQ_A_Neg_A_A3 RQ_P_Neg_A_A3 RQ_C_Neg_A_A3

RQ_T_Neg_A_A3 RQ_S_Neg_A_A3 RQ_F_Neg_A_A3 RQ_K_Neg_A_A3

RQ_PS_Neg_A_A3 RQ_ST_Neg_A_A3 RQ_EVT_Neg_A_A3

/FORMAT=AVALUE TABLES

/STATISTICS=CHISQ PHI

/CELLS=COUNT

/COUNT ROUND CELL.

FILTER OFF.

*KENNEN ZORGVERLENER, LEIDING BEVALLING.

SORT CASES BY OV_Kza_M_Cat2 (A).

SPLIT FILE BY OV_Kza_M_Cat2.

FREQUENCIES VARIABLES=RQ_R_Dom_A RQ_A_Dom_A RQ_P_Dom_A RQ_C_Dom_A RQ_T_Dom_A RQ_S_Dom_A RQ_F_Dom_A RQ_K_Dom_A RQ_PS_Dom RQ_ST_Dom RQ_EvT_A

/STATISTICS=MEAN MEDIAN

/NTILES 4

/ORDER=ANALYSIS.

FREQUENCIES RQ_R_Neg_A_A3 RQ_A_Neg_A_A3 RQ_P_Neg_A_A3 RQ_C_Neg_A_A3

RQ_T_Neg_A_A3 RQ_S_Neg_A_A3 RQ_F_Neg_A_A3 RQ_K_Neg_A_A3

RQ_PS_Neg_A_A3 RQ_ST_Neg_A_A3 RQ_EVT_Neg_A_A3.

SPLIT FILE OFF.

NPTESTS

/INDEPENDENT TEST (RQ_R_Dom_A RQ_A_Dom_A RQ_P_Dom_A RQ_C_Dom_A RQ_T_Dom_A RQ_S_Dom_A RQ_F_Dom_A RQ_K_Dom_A RQ_PS_Dom_A RQ_ST_Dom_A RQ_EvT_A) GROUP (OV_Kza_M_Cat2)

/MISSING SCOPE=ANALYSIS USERMISSING=EXCLUDE

/CRITERIA ALPHA=0.05 CILEVEL=95.

CROSSTABS

/TABLES=OV_Kza_M_Cat2 BY RQ_R_Neg_A_A3 RQ_A_Neg_A_A3 RQ_P_Neg_A_A3 RQ_C_Neg_A_A3

RQ_T_Neg_A_A3 RQ_S_Neg_A_A3 RQ_F_Neg_A_A3 RQ_K_Neg_A_A3

RQ_PS_Neg_A_A3 RQ_ST_Neg_A_A3 RQ_EVT_Neg_A_A3

/FORMAT=AVALUE TABLES

/STATISTICS=CHISQ PHI

/CELLS=COUNT

/COUNT ROUND CELL.

*URBANISATIE.

SORT CASES BY ZB_Urb_M (A).

SPLIT FILE BY ZB_Urb_M.

FREQUENCIES VARIABLES=RQ_R_Dom_A RQ_A_Dom_A RQ_P_Dom_A RQ_C_Dom_A RQ_T_Dom_A RQ_S_Dom_A RQ_F_Dom_A RQ_K_Dom_A RQ_PS_Dom RQ_ST_Dom RQ_EvT_A

/STATISTICS=MEAN MEDIAN

/NTILES 4

/ORDER=ANALYSIS.

FREQUENCIES RQ_R_Neg_A_A3 RQ_A_Neg_A_A3 RQ_P_Neg_A_A3 RQ_C_Neg_A_A3

RQ_T_Neg_A_A3 RQ_S_Neg_A_A3 RQ_F_Neg_A_A3 RQ_K_Neg_A_A3

RQ_PS_Neg_A_A3 RQ_ST_Neg_A_A3 RQ_EVT_Neg_A_A3.

SPLIT FILE OFF.

NPTESTS

/INDEPENDENT TEST (RQ_R_Dom_A RQ_A_Dom_A RQ_P_Dom_A RQ_C_Dom_A RQ_T_Dom_A RQ_S_Dom_A RQ_F_Dom_A RQ_K_Dom_A RQ_PS_Dom_A RQ_ST_Dom_A RQ_EvT_A) GROUP (ZB_Urb_M)

/MISSING SCOPE=ANALYSIS USERMISSING=EXCLUDE

/CRITERIA ALPHA=0.05 CILEVEL=95.

CROSSTABS

/TABLES=ZB_Urb_M BY RQ_R_Neg_A_A3 RQ_A_Neg_A_A3 RQ_P_Neg_A_A3 RQ_C_Neg_A_A3

RQ_T_Neg_A_A3 RQ_S_Neg_A_A3 RQ_F_Neg_A_A3 RQ_K_Neg_A_A3

RQ_PS_Neg_A_A3 RQ_ST_Neg_A_A3 RQ_EVT_Neg_A_A3

/FORMAT=AVALUE TABLES

/STATISTICS=CHISQ PHI

/CELLS=COUNT

/COUNT ROUND CELL.

*KANTOORTIJDEN.

SORT CASES BY ZB_Kan_M_Cat2 (A).

SPLIT FILE BY ZB_Kan_M_Cat2.

FREQUENCIES VARIABLES=RQ_R_Dom_A RQ_A_Dom_A RQ_P_Dom_A RQ_C_Dom_A RQ_T_Dom_A RQ_S_Dom_A RQ_F_Dom_A RQ_K_Dom_A RQ_PS_Dom RQ_ST_Dom RQ_EvT_A

/STATISTICS=MEAN MEDIAN

/NTILES 4

/ORDER=ANALYSIS.

FREQUENCIES RQ_R_Neg_A_A3 RQ_A_Neg_A_A3 RQ_P_Neg_A_A3 RQ_C_Neg_A_A3

RQ_T_Neg_A_A3 RQ_S_Neg_A_A3 RQ_F_Neg_A_A3 RQ_K_Neg_A_A3

RQ_PS_Neg_A_A3 RQ_ST_Neg_A_A3 RQ_EVT_Neg_A_A3.

SPLIT FILE OFF.

NPTESTS

/INDEPENDENT TEST (RQ_R_Dom_A RQ_A_Dom_A RQ_P_Dom_A RQ_C_Dom_A RQ_T_Dom_A RQ_S_Dom_A RQ_F_Dom_A RQ_K_Dom_A RQ_PS_Dom_A RQ_ST_Dom_A RQ_EvT_A) GROUP (ZB_Kan_M_Cat2)

/MISSING SCOPE=ANALYSIS USERMISSING=EXCLUDE

/CRITERIA ALPHA=0.05 CILEVEL=95.

CROSSTABS

/TABLES=ZB_Kan_M_Cat2 BY RQ_R_Neg_A_A3 RQ_A_Neg_A_A3 RQ_P_Neg_A_A3 RQ_C_Neg_A_A3

RQ_T_Neg_A_A3 RQ_S_Neg_A_A3 RQ_F_Neg_A_A3 RQ_K_Neg_A_A3

RQ_PS_Neg_A_A3 RQ_ST_Neg_A_A3 RQ_EVT_Neg_A_A3

/FORMAT=AVALUE TABLES

/STATISTICS=CHISQ PHI

/CELLS=COUNT

/COUNT ROUND CELL.

*REISTIJD.

SORT CASES BY ZB_Ret_M_Cat2 (A).

SPLIT FILE BY ZB_Ret_M_Cat2.

FREQUENCIES VARIABLES=RQ_R_Dom_A RQ_A_Dom_A RQ_P_Dom_A RQ_C_Dom_A RQ_T_Dom_A RQ_S_Dom_A RQ_F_Dom_A RQ_K_Dom_A RQ_PS_Dom RQ_ST_Dom RQ_EvT_A

/STATISTICS=MEAN MEDIAN

/NTILES 4

/ORDER=ANALYSIS.

FREQUENCIES RQ_R_Neg_A_A3 RQ_A_Neg_A_A3 RQ_P_Neg_A_A3 RQ_C_Neg_A_A3

RQ_T_Neg_A_A3 RQ_S_Neg_A_A3 RQ_F_Neg_A_A3 RQ_K_Neg_A_A3

RQ_PS_Neg_A_A3 RQ_ST_Neg_A_A3 RQ_EVT_Neg_A_A3.

SPLIT FILE OFF.

NPTESTS

/INDEPENDENT TEST (RQ_R_Dom_A RQ_A_Dom_A RQ_P_Dom_A RQ_C_Dom_A RQ_T_Dom_A RQ_S_Dom_A RQ_F_Dom_A RQ_K_Dom_A RQ_PS_Dom_A RQ_ST_Dom_A RQ_EvT_A) GROUP (ZB_Ret_M_Cat2)

/MISSING SCOPE=ANALYSIS USERMISSING=EXCLUDE

/CRITERIA ALPHA=0.05 CILEVEL=95.

CROSSTABS

/TABLES=ZB_Ret_M_Cat2 BY RQ_R_Neg_A_A3 RQ_A_Neg_A_A3 RQ_P_Neg_A_A3 RQ_C_Neg_A_A3

RQ_T_Neg_A_A3 RQ_S_Neg_A_A3 RQ_F_Neg_A_A3 RQ_K_Neg_A_A3

RQ_PS_Neg_A_A3 RQ_ST_Neg_A_A3 RQ_EVT_Neg_A_A3

/FORMAT=AVALUE TABLES

/STATISTICS=CHISQ PHI

/CELLS=COUNT

/COUNT ROUND CELL.

*VSV GROOTTE.

SORT CASES BY VSV_GR_M (A).

SPLIT FILE BY VSV_GR_M.

FREQUENCIES VARIABLES=RQ_R_Dom_A RQ_A_Dom_A RQ_P_Dom_A RQ_C_Dom_A RQ_T_Dom_A RQ_S_Dom_A RQ_F_Dom_A RQ_K_Dom_A RQ_PS_Dom RQ_ST_Dom RQ_EvT_A

/STATISTICS=MEAN MEDIAN

/NTILES 4

/ORDER=ANALYSIS.

FREQUENCIES RQ_R_Neg_A_A3 RQ_A_Neg_A_A3 RQ_P_Neg_A_A3 RQ_C_Neg_A_A3

RQ_T_Neg_A_A3 RQ_S_Neg_A_A3 RQ_F_Neg_A_A3 RQ_K_Neg_A_A3

RQ_PS_Neg_A_A3 RQ_ST_Neg_A_A3 RQ_EVT_Neg_A_A3.

SPLIT FILE OFF.

NPTESTS

/INDEPENDENT TEST (RQ_R_Dom_A RQ_A_Dom_A RQ_P_Dom_A RQ_C_Dom_A RQ_T_Dom_A RQ_S_Dom_A RQ_F_Dom_A RQ_K_Dom_A RQ_PS_Dom_A RQ_ST_Dom_A RQ_EvT_A) GROUP (VSV_GR_M)

/MISSING SCOPE=ANALYSIS USERMISSING=EXCLUDE

/CRITERIA ALPHA=0.05 CILEVEL=95.

CROSSTABS

/TABLES=VSV_GR_M BY RQ_R_Neg_A_A3 RQ_A_Neg_A_A3 RQ_P_Neg_A_A3 RQ_C_Neg_A_A3

RQ_T_Neg_A_A3 RQ_S_Neg_A_A3 RQ_F_Neg_A_A3 RQ_K_Neg_A_A3

RQ_PS_Neg_A_A3 RQ_ST_Neg_A_A3 RQ_EVT_Neg_A_A3

/FORMAT=AVALUE TABLES

/STATISTICS=CHISQ PHI

/CELLS=COUNT

/COUNT ROUND CELL.
